# CO_2_ Elevation Accelerates Phenology and Alters Carbon/Nitrogen Metabolism *vis-à-vis* ROS Abundance in Bread Wheat

**DOI:** 10.3389/fpls.2020.01061

**Published:** 2020-07-17

**Authors:** Birendra K. Padhan, Lekshmy Sathee, Hari S. Meena, Sandeep B. Adavi, Shailendra K. Jha, Viswanathan Chinnusamy

**Affiliations:** ^1^Division of Plant Physiology, ICAR-Indian Agricultural Research Institute, New Delhi, India; ^2^Division of Genetics, ICAR-Indian Agricultural Research Institute, New Delhi, India

**Keywords:** CO_2_ elevation (CE), high affinity nitrate transporters (HATS), nitrosothiols, reactive oxygen species (ROS), reactive nitrogen species (RNS), C/N ratio, nitrogen use efficiency (NUE)

## Abstract

Wheat is an important staple food crop of the world and it accounts for 18–20% of human dietary protein. Recent reports suggest that CO_2_ elevation (CE) reduces grain protein and micronutrient content. In our earlier study, it was found that the enhanced production of nitric oxide (NO) and the concomitant decrease in transcript abundance as well as activity of nitrate reductase (NR) and high affinity nitrate transporters (HATS) resulted in CE-mediated decrease in N metabolites in wheat seedlings. In the current study, two bread wheat genotypes Gluyas Early and B.T. Schomburgk differing in nitrate uptake and assimilation properties were evaluated for their response to CE. To understand the impact of low (LN), optimal (ON) and high (HN) nitrogen supply on plant growth, phenology, N and C metabolism, ROS and RNS signaling and yield, plants were evaluated under short term (hydroponics experiment) and long term (pot experiment) CE. CE improved growth, altered N assimilation, C/N ratio, N use efficiency (NUE) in B.T. Schomburgk. In general, CE decreased shoot N concentration and grain protein concentration in wheat irrespective of N supply. CE accelerated phenology and resulted in early flowering of both the wheat genotypes. Plants grown under CE showed higher levels of nitrosothiol and ROS, mainly under optimal and high nitrogen supply. Photorespiratory ammonia assimilating genes were down regulated by CE, whereas, expression of nitrate transporter/*NPF* genes were differentially regulated between genotypes by CE under different N availability. The response to CE was dependent on N supply as well as genotype. Hence, N fertilizer recommendation needs to be revised based on these variables for improving plant responses to N fertilization under a future CE scenario.

## Introduction

Atmospheric CO_2_ concentration is increasing exponentially and from the preindustrial level, it has increased by 40% and is expected to reach approximately 935 ppm by the end of 2100 (IPCC, 2014). From the CO_2_ enrichment studies, it is evident that CO_2_ elevation (CE) can impart major changes such as declined gas exchange rate and photorespiration, while maintaining the higher net CO_2_ assimilation rate (Ainsworth and Long, 2005; Long et al., 2006; Ainsworth et al., 2008), improved water use efficiency (Drake et al., 1997; Leakey, 2009), increased dry matter accumulation and grain yield (Ziska, 2008) in C_3_ crops like wheat. Reports suggest that plants grown under CE have higher photosynthetic activity, higher nutrient uptake and increased productivity (Gruda et al., 2019). Photosynthetic acclimation of plants in CE has been shown in different studies (Zhu et al., 2012; Tausz-Posch et al., 2013) and there is a close relationship between photosynthesis, biomass production and yield (Kellner et al., 2019; Pastore et al., 2019). CO_2_ enrichment increases the biomass of food crops and tree crops (Luo et al., 2004; Lam et al., 2012) but lead to reduced food quality especially N constituents (Cotrufo et al., 1998; Taub and Wang, 2008). In the past, the lower N content of CE grown plants/food products has been credited to dilution effect of carbon accumulation (Taub and Wang, 2008), or low N availability (Rogers et al., 1998) or inhibition of shoot N assimilation (Bloom et al., 2002a; Bloom et al., 2002b) or decreased rate of transpiration (Myers et al., 2014). However, there are contradictory reports: high nitrate accumulating plants, cucumber and barley performed well in CE even with nitrate supply larger than 10 mM (Bloom et al., 2010; Asensio et al., 2015). Inhibition of nitrate assimilation is more significant in leaves as the competition for reductant and the hindrances in accessibility of nitrate are higher in leaves (Hawkesford, 2012). The reduced transpiration rates under CE indirectly inhibit N uptake and transport to leaves (Correia et al., 2005; Jauregui et al., 2016).

Besides the fact that N limitation is one of the factors determining the enhancement in plant growth under CE (Rogers et al., 1998), the form of N supplied also has major bearing on uptake kinetics of N (Hirel et al., 2007; Xu et al., 2012), growth rate and N assimilation (Leakey, 2009). Experimental evidences confirm that CO_2_ elevation decreases the N uptake rate when nitrate was the N source but uptake rate was unaltered when ammonium was the N source (Rachmilevitch et al., 2004; Asensio et al., 2015). On the other hand, when either nitrate or ammonium was the sole N source, plant growth, metabolism as well as photosynthesis differed significantly in *Arabidopsis*, tomatoes and wheat under CE (Leakey, 2009; Asensio et al., 2015). Plants sustain the N limited condition by restricting the investment of N to leaves and diverting it to roots to maintain optimal N distribution and attain higher N use efficiency (NUE) (Zhang et al., 2017). However, CE mediated stimulation in growth of aboveground plant parts was miniscule when the cost of N acquisition was high and *vice versa* (Terrer et al., 2018).

Previous studies suggest a decline in carotenoid content in leaves of different plant species (Dhami et al., 2018). From meta-analysis it was found that CE decreases plant carotenoid concentration by 15% (Loladze et al., 2019). Even though CE is expected to reduce production of reactive oxygen species (ROS) in C_3_ plants, Qiu et al. (2008) observed increased abundance of leaf protein carbonylation, a potent marker of oxidative stress in *Arabidopsis* and soybean plants exposed to CE. Qiu et al. (2008) also suggested an increased abundance of ROS in plants grown under CE, based on higher abundance of cytosolic *APX1* that is transcriptionally regulated by oxidative stress and plays key role in ROS removal. Cheeseman (2006) found that CE grown leaves of soybean showed higher abundance of H_2_O_2_ as compared with CA grown plants. It is speculated that interaction of bicarbonate with iron or heme groups triggers ROS generation in CE conditions (Arai et al., 2005). *In silico* gene expression data (Miyazaki et al., 2004; Li et al., 2006) also indicated that CE up-regulates many genes associated with redox control and thus impairment in ROS balance. Recently, Foyer and Noctor (2020) also concluded that apoplastic ROS signals were increased at high CO_2_, and possibly due to the involvement NADPH oxidases (Cheeseman, 2006; Qui et al., 2008; Mhamdi and Noctor, 2016). Although CE helps in the retention of chlorophyll pigment (Li et al., 2019), it might also lead to production of toxic ROS and RNS (reactive nitrogen species) like nitric oxide (NO) (Adavi and Sathee, 2018; Foyer and Noctor, 2020).

CO_2_ Elevation results in production of NO *via* nitric oxide synthase (NOS) pathway and regulates the NR activity (Du et al., 2016). Regulation of NR activity by NO is dependent on nitrate availability: low nitrate leads to up-regulation of NR activity whereas; high nitrate leads to downregulation of NR activity (Du et al., 2008; Adavi and Sathee, 2018). Reduced NR activity under CE is due to S-nitrosylation mechanism by NO (Du et al., 2016). The sustained positive effect of CE on plant growth and yield requires modifications in other environmental variables (temperature, light quality) and appropriate nutrient management (Pan et al., 2019; Kumari et al., 2019).

The aims of this study were to understand 1) the response of two diverse wheat genotypes to CE, 2) changes in ROS and RNS accumulation under CE, and 3) impact of CE on expression of genes associated with N metabolism, and ROS and RNS balance. To achieve these aims, two separate experiments were conducted under hydroponics (Experiment I) and pot culture (experiment II) with two wheat genotypes Gluyas Early (V1), B.T. Schomburgk (V2) which are diverse in terms of nitrate uptake and assimilation capacities (**Supplementary Files 1A, B**). These experiments were laid out in Plant growth chambers at National Phytotron Facility, ICAR-IARI, New Delhi.

## Methodology

### Growing Conditions

Experiments were laid out in growth chambers (Model: PGW 36, Conviron, Winnipeg, Canada) at National Phytotron Facility, ICAR-IARI, New Delhi. One set of plants were maintained CO_2_ ambient (400 ± 10 µl/l or ppm, CA) and another set at CO_2_ elevation (700 ± 10 µl/l or ppm, CE) with temperature of 22°C/12°C (day/night), 80–90% of RH (relative humidity), 400 μmol m^−2^ s^−1^ of PFD (photon flux density, PAR) and 10 h of photoperiod. IRGA LI-6400, (LICOR, Lincoln, NE, USA, infrared gas analyzer) was used to check the CO_2_ level regularly. The investigation consisted of two different experiments (**Supplementary File 2**).

### Experiment I: Hydroponics Study

Wheat seedlings were raised in hydroponics as described in Adavi and Sathee (2018). Seeds were washed with double distilled water and then surface sterilized with 0.1% mercuric chloride (HgCl_2_) for 5 min. To remove the traces of HgCl_2_, seeds were thoroughly washed for five to six times with double distilled water. The surface sterilized seeds were germinated in Petri plates lined with moist germination paper and uniform seedlings were transplanted into trays containing nitrogen free Hoagland solution after 5–6 days of germination (Hoagland and Arnon, 1950) with three nitrogen treatments, i.e., low (LN, 0.05 mM NO_3_^-^), optimum (ON, 5 mM NO_3_^-^) and high (HN, 20 mM NO_3_^-^). The plants were held on Styrofoam sheets in plastic trays holding10 L of nutrient solution prepared in sterile deionized water. The solution was aerated continuously through aquarium pumps. Growing media was prepared using sterile deionized water and media was changed after every 2–3 days to maintain axenic culture conditions and also to ensure steady supply of nutrients. Samples for recording physiological, biochemical and molecular observations were done after 35 days of transplanting (35DAT). Three independent biological replications were maintained for each of the treatments and all the parameters were estimated in triplicates.

#### Estimation of Biomass and Leaf Area

Plants were harvested from each of the three biological replications, shoot and root fresh weight was recorded immediately after harvest of sample. Dry weight of tissues was recorded once they attained the constant weight after drying in oven at 60 °C. Leaf area meter (LiCOR 3100, Lincoln Nebraska, USA) was used to record leaf area.

#### Analysis of Root Traits

For recording root traits, roots of representative plants were taken for each replication were scanned in a root scanner (Epson, Expression 11000XL, Graphic Art Model) and scanning was done in triplicates for each treatment. Scanned root images were analyzed by using Win-RHIZO, Regent Instruments to calculate different root parameters. Based on diameter of the root, roots were categorized as main roots (diameter >0.5mm) and lateral roots (diameter ≤0.5mm). Different traits like length, volume, surface area and diameter of root were retrieved. Two representative plants were taken per replication and scanning was done in three such biological replicates in each treatment.

#### Estimation of Plant Pigments

Chlorophyll and carotenoid contents were determined according to Hiscox and Israelstam (1979). Freshly sampled, uppermost fully expanded leaves were used for the estimation. Excluding the midrib, the leaves were fragmented into small pieces of around 2 mm size. These fragments were mixed thoroughly and 25 mg of this sample were placed inside the test tubes containing 5 ml of DMSO (dimethyl sulphoxide). The test tubes were incubated in dark for 4 h at 65°C in oven which facilitate the chlorophyll extraction into the solution. The absorbance was measured at 470, 645 and 663 nm using UV–visible spectrophotometer (Model Specord Bio-200, AnalytikJena, Germany). Total chlorophyll and total carotenoids contents were calculated according to Arnon (1949) and expressed as mg g^−1^DW.

#### Estimation of Nitrate Reductase (NR) Activity in Plant Tissues

Fresh shoot and root samples weighing 0.5 g were homogenized using pre-chilled mortar and pestle in 5 ml of cold extraction buffer (phosphate buffer 0.1 M, pH 7.5 containing 5-mM EDTA and 5-mM cysteine). The homogenate is centrifuged at 10,000 rpm for 15 min at 4°C (Hageman and Huckles by, 1971). The supernatant was aliquoted into a new centrifuge tube and kept on ice until enzyme assay was carried out. The assay mixtures (test sample and blank sample) in a final volume of 3 ml were prepared in separate test tubes containing 1,900 µl of phosphate buffer (0.1 M, pH 7.5) and 500 µl of 0.1-M KNO_3_ and100 µl of 10-mM NADH. The enzymatic reaction was initiated by the addition of 500 µl of enzyme extract into the assay mixture. As for the blank samples, the enzyme extracts were substituted with 500 μl of deionized water. The mixtures were incubated at 30°C for 30 min. Reaction was terminated by adding 0.2 ml of 1 M zinc acetate, followed by 1.8 ml of 75% ethanol. The precipitate formed is removed by centrifugation at 2,000 rpm for 5 min at room temperature. Subsequently, 1 ml of 1% (w/v) sulfanilamide solution and 1 ml of 0.02% (w/v) *N*-(1-naphthyl) ethylene diamine solution were added to the supernatant (Klepper et al., 1971). Absorbance was recorded after 20 min against blank sample at 540 nm. The enzyme activity is expressed as µmol nitrite formed mg^−1^ protein h^−1^.

#### Estimation of GS, GOGAT, GDH Activity

Leaf and root samples were separately used for extraction and assay of enzymes, GS (Glutamine synthetase), GOGAT (Glutamine oxoglutarate aminotransferase) and GDH (Glutamate dehydrogenase) were done following Mohanty and Fletcher (1980). Leaf and root samples were extracted in Tris–HCl buffer, which contains 100 mM Tris–HCl, 100 mM sucrose, 10 mM EDTA and 10 mM MgCl_2_. Extracts were centrifuged (sigma 3K30) at 5,000*g* for 10 min at 4°C. Supernatant was collected and re-centrifuged at 12,000*g* for 15 min at 4°C. Supernatant obtained, after second round of centrifugation, was used for assay of GS and GOGAT. Pellet was dissolved in 50 mM phosphate buffer (pH 7.5) containing 2.14 g/100 ml sucrose and was used for assay of GDH. For assaying Gs activity, reaction mixture consisting of 75 mM Tris buffer, 50 mM MgSO_4_, 5 mM cysteine, 125 mM α-glutamate, 5 mM ATP and 10 mM hydroxylamine along with 0.2 ml of enzyme extract was incubated for 30 min at 37°C. To stop the reaction 0.5 ml FeCl_3_ reagent was added followed by centrifugation at 1,500–2,000*g* for 10 min. Absorbance was measured using a UV–visible spectrophotometer (Specord Bio-200, AnalytikJena, Germany). Activity of GS was expressed as µmol γ-glutamyl hydroxamate formed g^−1^ protein h^−1^.

GOGAT and GDH activity was measured by estimating µmol NADH oxidized g^−1^ protein h^−1^. To assay GOGAT activity, reaction mixture containing 75 mM Tris–HCl, 10 mM α-ketoglutaric acid 40 mM L-glutamine, 0.1 ml of enzyme extract was prepared. Required amount of 1.5 mM of NADH was added to the reaction mixture prior recording absorbance at 340 nm for 60 s. For assaying GDH activity, reaction mixture containing 75 mM phosphate buffer, 20 mM α-ketoglutaric acid and 300 mM NH_4_Cl and 0.2 ml of enzyme extract was prepared. Required amount of 1.5 mM of NADH was added to the reaction mixture prior recording absorbance at 340 nm for 60 s.

Soluble protein content of tissue extracts were determined to express enzyme activities in terms of unit amount of protein. Adequate amount of plant extract was mixed with 2 ml of Coomassie Brilliant Blue G-250 (CBB, Biotechnology grade, Genetix brand) followed by vortexing. After 2-minute of incubation at room temperature, absorbance was measured at 595 nm using UV–visible spectrophotometer (Specord Bio-200, AnalytikJena, Germany).

#### Estimation of S-Nitrosothiols

S-nitrosothiol content in leaves and roots were estimated as described in Arc et al. (2013). Approximately, 0.5 g of plant sample was ground in ice cold extraction buffer (25 mM Hepes-NaOH, containing 1 mM M EDTA, pH 7.8) followed by centrifugation at 12,500rpm for 25 min at 4°C. For estimating S-nitrosothiols, appropriate amount of extract was mixed with 50 µl ammonium sulfamate (0.5%). The reaction mixture was incubated at room temperature for 2 min followed by addition of 300 µl of sulfanilamide (7% in 1NHCl) and 300 µl of NEDD (0.1%). After vortexing, the samples were incubated for 30 min at room temperature. Care was taken to maintain darkness, throughout the assay procedure. Absorbance of the sample was recorded at 540 nm using UV–Visible spectrophotometer (model: Specord Bio-200, AnalytikJena, Germany).

#### Estimation of Soluble Sugar and Starch Content

Adequate amount of plant samples (oven dried and powdered) were extracted four times in boiling 20 ml of 80% (v/v) ethanol for 4–5 min (McCready et al., 1950). The volume of supernatant was made up to 100 ml with double distilled water in volumetric flask. Required amount of aliquot was used to determine total soluble sugars using anthrone reagent (Sadasivam and Manickam, 1996). To 1 ml of sugar sample, 4 ml solution of anthrone reagent (100 mg anthrone was dissolved in 100 ml chilled concentrated H_2_SO_4_) was added and the mixture was heated on a boiling water bath for 8 min followed by cooling. The optical density of green to dark green color was recorded at 630 nm in UV–visible spectrophotometer (model: Specord Bio-200, AnalytikJena, Germany). A blank and two freshly prepared glucose standards were also included with each set of sample. For determining starch content, residue left over after extraction of sugars was dried, powdered and hydrolyzed in a glycerine bath with 10 ml of 1N HCl at 112–115°C for 30 min. Samples were allowed to cool and repeatedly washed with double distilled water till negative results were obtained for iodine test. Once negative results were confirmed, the extracts were pooled and volume was made up to 100 ml. An aliquot was used to determine the total sugar following anthrone reagent method. Starch content was calculated by multiplying the glucose values (1 OD = 600 µg g^−1^ fresh wt.) with 0.9 (Pucher et al., 1948).

#### Estimation of Tissue Nitrate Content

Tissue nitrate content was estimated by the method described by Downes (1978). Oven dried samples were powdered (20 mesh) and 100 mg of this sample along with nitrate free charcoal of equivalent weight (100 mg) were transferred to conical flask containing 15 ml of double distilled water. The samples were mixed thoroughly and boiled for 3–4 min and filtered through Whatman filter paper-42. The residue left over was re-extracted with double distilled water and volume made up to 50 ml. After 20–30 min nitrate content was estimated by measuring the absorbance at 540 nm.

#### Estimation of Total Free Amino Acid Content

Ninhydrin test was used to determine the content of free amino acids as described by Sircelj et al. (2005). Oven dried samples were powdered and extracted in 80% ethanol and extract was centrifuged at 8,000*g* for 15 min. The reaction mixture consisted of supernatant and ninhydrin reagent which was vortexed vigorously and incubated in a boiling water bath for 15 min followed by addition of diluent (50% v/v ethanol). Samples were allowed to cool to room temperature and absorbance was measured at 570 nm.

#### Estimation of Reactive Oxygen Species

**Tissue localization of superoxide ions (O_2_**^−^**) and** hydrogen peroxide (H_2_O_2_) were determined in physiologically active, second leaf of wheat seedlings (Kumar et al., 2014). For estimating **O_2_**^−^ ions, **leaves were** cut into 1 cm long fragments and immediately dipped in 6 mM NBT solution prepared in sodium citrate buffer (pH 7.5) and infiltrated using vacuum pump for 10 min at 60 KPa and followed by incubation for 10 min at room temperature. After incubation, samples were immersed in 80% ethanol and heated in boiling water bath till the chlorophyll from the tissue was cleared off. After cooling samples were dipped in 20% glycerol and mounted on glass slides carefully. Development of dark blue color indicated the presence of O_2_^−^. For detection of H_2_O_2_, leaf segments were immediately dipped into DAB (3,3′-Diaminobenzidine) solution (1 mg/ml) with pH 3.8 in a petri dish (35 mm) using tweezers. Vacuum infiltration of dipped samples were done at 60 KPa pressure for 10 min and illuminated at room temperature for 10 min. After incubation, chlorophyll pigments were cleared off by transferring samples to 80% ethanol and heating in boiling water bath. Samples were cooled and dipped in 20% glycerol and mounted on glass slides. Appearance of brown colored product confirmed the presence of H_2_O_2_. Slides were visualized and images were captured using a stereo microscope (EVOS XL Core).

The spectrophotometric assay of total superoxide radical content in the tissues is based on the principle of formation of blue colored formazone by nitroblue tetrazolium chloride with superoxide radicals (O_2_^−^) by inhibiting total superoxide dismutase (SOD) activity, as described by Chaitanya and Naithani (1994). For the assay plant material was collected and flash frozen in liquid Nitrogen and stored in −80°C freezer was used.

#### Expression Profiling of Nitrate Uptake and Assimilation Genes

Impact of N (LN—0.05mM NO_3_^−^, ON—5mM NO_3_^−^, HN—20mM NO_3_^−^) levels and CO_2_ concentrations (CA: 400 ± 10 µl/l and CE: 700 ± 10 µl/l)) on expression of genes associated with nitrogen metabolism (low affinity nitrate transporters; NPF) and N assimilation genes were done in leaves and roots of wheat genotypes. Expression of the following genes: *TaNPF1.1*, *TaNPF2.1*, *TaNPF2.2*, *TaNPF2.3*, *TaNPF2.4*, *TaNPF6.1*, *TaNPF6.2*, *TaNPF6.5*, *TaNPF6.6*, *TaNPF7.1*, *TaGS2* and *Ta Fd-GOGAT* were studied. The procedure for plant growth and expression profiling was done as described in Adavi and Sathee (2018). qRT-PCR was done using gene specific primers (**Supplementary File 3**). Melt curve data collection and analysis was enabled. To confirm the specificity of the primers agarose gel electrophoresis of qRT-PCR products were done. *TaActin* (Traes_1AL_E195290EF) was used as a house keeping gene and used to normalize the data (Lekshmy and Jha, 2017).

#### *In-Silico* Expression Analysis of Genes Encoding Antioxidant Enzymes NCBI-GEO

Analysis of publically available transcriptome data was used to decipher expression response of different antioxidant enzymes in response to CE. Expression data of relevant experiments GSE48620 (study comparing the global gene expression of *Triticum aestivum* cv Norstar grown either under CA or CE) was downloaded from NCBI-GEO (https://www.ncbi.nlm.nih.gov/gds) and analyzed to study *gene* expression.

### Experiment II: Pot Culture Study

The experiment was carried out in climate-controlled growth chambers (Model: PGW 36, Conviron, Winnipeg, Canada) at National Phytotron facility, ICAR-IARI, New Delhi. Sowing was done in plastic pots measuring 15 cm in diameter and 20 cm in height. After three leaf stage thinning was done and only four plants were allowed to grow in each pot. From sowing, half of the plants (20 pots) were grown in a chamber with 400 ± 10 µl/l [CO_2_] (CA), and another half were grown in chamber with 700 ± 10 µl/l [CO_2_] (CE) concentration. Each pot was filled with 3 kg growing media with composition of vermiculite, sand and coco pit in 1:2:1 ratio. The media contained approximately 1.0 g kg^−1^ of total N. Nitrogen was applied in the form of KNO_3_. In each chamber, 1.5 g N was applied to half of the pots (ON, optimal N treatment), while 5 g N was applied into the other half (HN, high N treatment). Completely randomized design was used for the experiment with 10 replicates for each treatment.

#### Estimation of Plant Pigments and Assay of Enzymes

Chlorophyll and Carotenoid contents and activity of *in vitro* NR (nitrate reductase), GS (glutamine synthatase, γ-glutamylhydroxamate formed mg^−1^ protein h^−1^), GOGAT (glutamate synthase, µmol NADH oxidized g^−1^ protein h^−1^) and GDH (glutamate dehydrogenase, µmol NADH oxidized g^−1^ protein h^−1^) were determined spectrophotometrically at different growth stages viz., vegetative, booting, anthesis and grain filling.

#### Measurement of Gas Exchange Parameters

A LI-6400XTPortable Photosynthesis System (Model: LI-COR, Lincoln, Nebraska, USA) was used to record photosynthetic rate (PN, μmol CO_2_ m^−2^ s^−1^), stomatal conductance (Gs, mmol H_2_O m^−2^ s^−1^) and transpiration rate (Tr, mmol H_2_O m^−2^ s^−1^) at vegetative and anthesis stages of the crop.

#### Phenological Observations

Observations on date of booting, anthesis, milking, grain filling and physiological maturity were recorded by visual observation based on Feekes scale (Large, 1954).

#### Analysis of Yield Parameters

Ruler was used to measure the plant height which is expressed as cm plant^−1^. Sampling was done in triplicates with each replication having three plants. Plants were kept in oven at 60°C till the samples were dried and attain constant dry weight, subsequently dry weight was recorded. The yield parameters such as number of ears per plant, grains per ear, spikelets per ear, ear length (cm) etc. were also recorded.

#### Estimation of Nutrient Content

Carbon (C) and nitrogen (N) content in leaf tissues were estimated by using CHNS analyzer (Model: EURO EA elemental analyzer, Polo Tecnologico, Pavia). Oven dried moisture free plant samples (0.5 mg to 1.0 mg) were properly packed and weighed before estimating C and N content. N content in growing media was estimated before planting and after harvest by Kjeldahl’s method (Kjeldahl, 1883).

#### Statistical Analysis

Completely randomized design was used for laying out both the experiments. Data represent the mean of the three biological replications. F test was carried out to test the significance of the treatment differences and the least significant differences (LSD) were computed to test the significance of the different treatments at 5% level of probability by using SPSS 16.0.

## Results

### CE Enhances Biomass Accumulation

**Figure 1:**
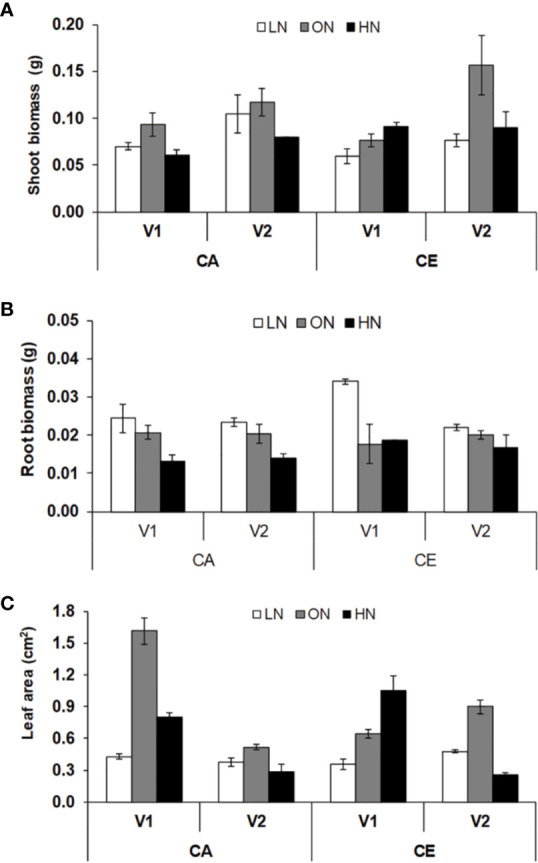
Interactive effect of CO_2_ elevation (CE, 700±10 µl/l, CO_2_ ambient :CA, 400±10 µl/l) and nitrogen availability (Low Nitrogen: LN, 0.05mM nitrate, Optimum Nitrogen: ON, 5mM nitrate, High Nitrogen: HN, 20mM nitrate) on shoot boimass **(A)**, root biomass **(B)** and leaf area **(C)** of 40 days old seedlings of wheat genotypes Gluyas Early (V1) and BT-Schomburgk (V2) raised in hydroponic culture. Values are mean (±SE) of 3 biological replicates.

Leaf area, shoot and root dry weight of seedlings were significantly different with regard to N level and CO_2_ treatments **(****Figure 1****)**. Genotypic differences were not significant in root biomass. All the interactive effects were significant for leaf area. The average values of shoot biomass were highest for ON treatment in both CA (0.105 g) and CE (0.117 g) treatments, whereas HN treatment resulted in reduction of shoot biomass in both CA (0.07 g) and CE (0.091 g) treatments. Irrespective of treatments B.T. Schomburgk (V2) always maintained higher shoot biomass **(****Figure 1A****)**. The average values of root biomass were highest for LN treatment in both CA (0.105 g) and CE (0.117 g) treatments, whereas HN treatment resulted in reduction of shoot biomass in both CA (0.024 g) and CE (0.028 g) treatments. Plants grown under CE displayed approximately 11% increase in root biomass than CA treatment **(****Figure 1B****)**. The average values of leaf area were highest for ON treatment in both CA (0.78) and CE (0.75) conditions, whereas HN treatment resulted in reduction of leaf area in both CA and CE treatments **(****Figure 1C****)**. Plants grown under CE displayed approximately 8% increase in root biomass as compared to that under CA treatment.

### CE Changes Root Traits

**Figure 2:**
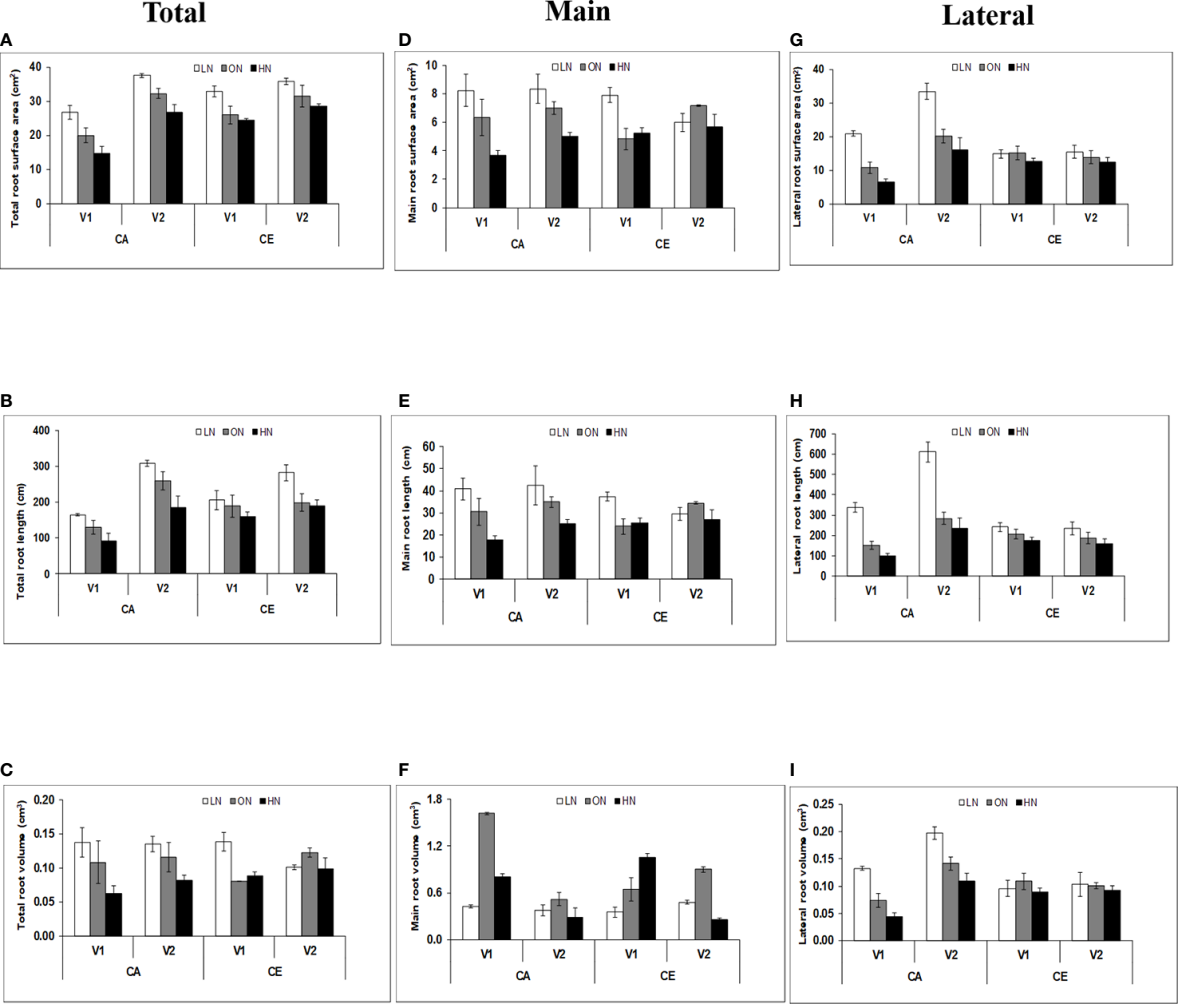
Interactive effect CO_2_ elevation (CE, 700±10 µl/l, CO_2_ ambient :CA, 400±10 µl/l) and nitrogen availability (Low Nitrogen: LN, 0.05mM nitrate, Optimum Nitrogen: ON, 5mM nitrate, High Nitrogen: HN, 20mM nitrate) on surface area (cm^2^) **(A, D, G)**, length (cm) **(B, E, H)** and volume (cm^3^) **(C, F, I)** of total, lateral and main roots of 40 days old seedlings of wheat genoytypes Gluyas Early (V1) and BT-Schomburgk (V2) raised in hydroponic culture. Values are mean (±SE) of 3 biological replicates.

Significant differences were observed in total root length, total root volume, average root diameter, total root surface area, length of main roots (diameter >0.5 mm), length of lateral roots (diameter ≤0.5 mm), volume of main roots, volume of lateral roots, surface area of main roots and surface area of lateral roots with various level of N and CO_2_. The total root surface area (cm^2^) of wheat seedlings was highest in LN followed by ON and HN, respectively, in both CA and CE **(****Figure 2****)**. Average values of total root surface area was higher in CE than in CA. B.T. Schomburgk (V2) showed 1.7% decrease in root surface area under CE than that in CA, whereas Gluyas Early (V1) showed 35% higher root surface area under CE than CA. Average values of main root surface area was higher in CE than CA. However, the changes in CE over CA were non-significant in both genotypes. V2 showed 20% decrease in lateral root surface area under CE than CA whereas V1 showed 12% higher lateral root surface area under CE than CA. The average root diameter was affected by both CO_2_ and N treatments. V2 showed 27% increase in average root diameter, while V1 showed only 2% higher average root diameter under CE as compared with CA. The effect of CE on total root length and main root length were non-significant, however, CO_2_ treatment resulted in significant alterations on lateral root length **(****Figure 2****)**. There was a significant difference among the genotypes also, V2 showed 1.1% decrease in lateral root length under CE than CA, whereas V1 showed 4.4% higher lateral root length under CE than CA. Under CE, the differences among total root volume and main root volume were non-significant. However, CO_2_ treatment resulted in significant alterations on lateral root volume **(****Figure 2****)**. There was a significant difference among the genotypes also, V2 showed 3.4% decrease in lateral root volume under CE than CA whereas V1 showed 1.8% higher lateral root volume under CE than CA.

### CE Resulted in Higher Rate of Photosynthesis and Reduces Gas Exchange

**Figure 3:**
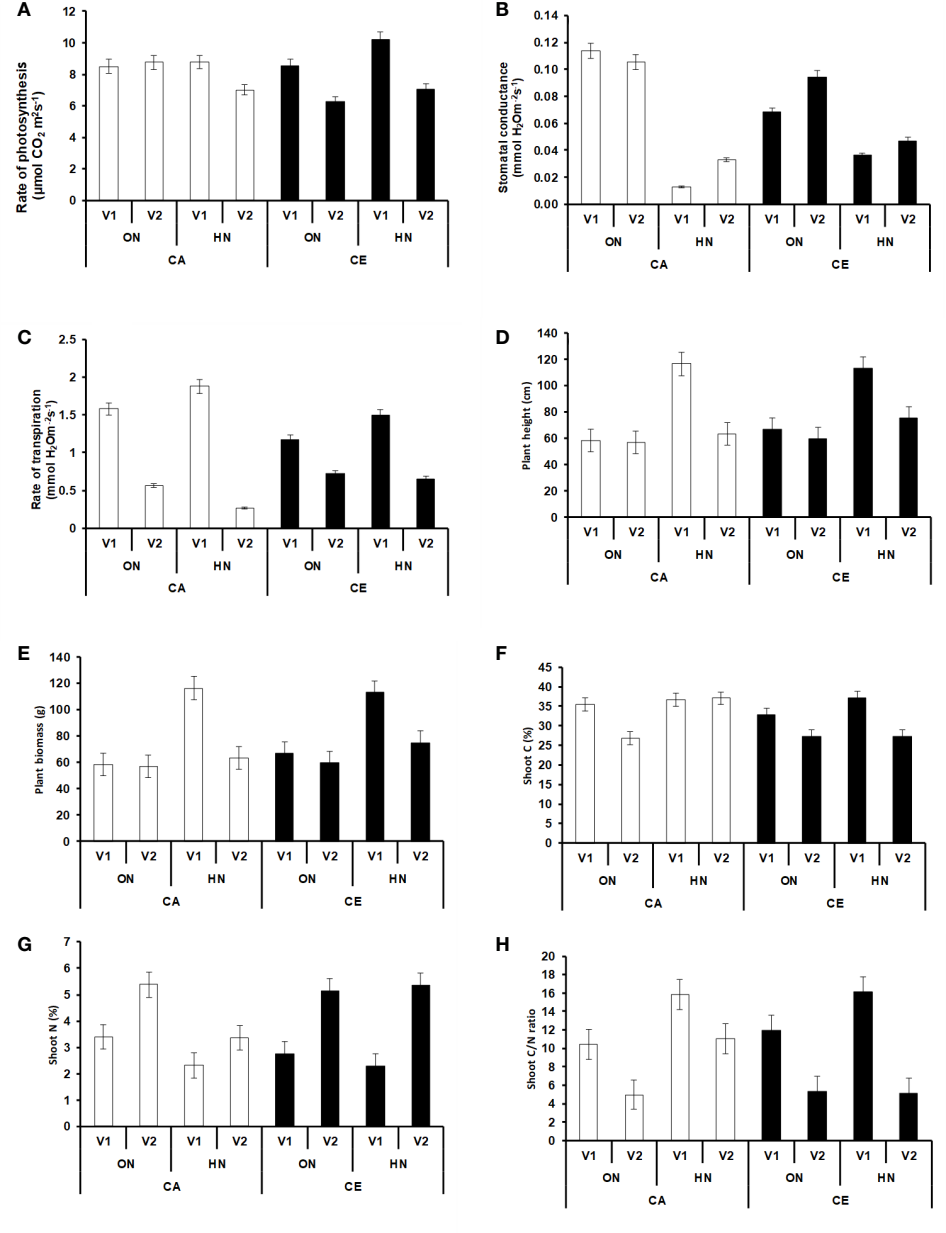
Intereactive effect of CO_2_ elevation (CE, 700±10 µl/l, CO_2_ ambient :CA, 400±10 µl/l) and Optimum Nitrogen (ON: 5mM nitrate) and High Nitrogen (HN: 20mM nitrate) availability on Photosynthetic parameters at anthesis **(A–C)** Plant height **(D)**, Plant biomass **(E)**, Shoot C (%) **(F)**, Shoot (N%) **(G)** and Shoot C/N ratio **(H)** at harvest in pot grown plants of wheat genotypes Gluyas Early (V1) and BT-Schomburgk (V2).Values are mean (±SE) of 3 biological replicates.

Plants grown under CE showed an enhanced rate of photosynthesis (2% increase over CA) at the vegetative stage **(****Figure 3A****)**. In the anthesis stage, the increase in photosynthetic rates was prominent in only in V2 plants (8% increase over CA). There was a general decline in both stomatal conductance and rate of transpiration in CE grown plants. The rate of photosynthesis was higher in vegetative stage as compared to anthesis, whereas stomatal conductance and rate of transpiration increased during the later stage **(****Figures 3B, C****)**.

### CE Promotes Growth, Increases C/N Ratio and Accelerates Phenology

**Figure 4:**
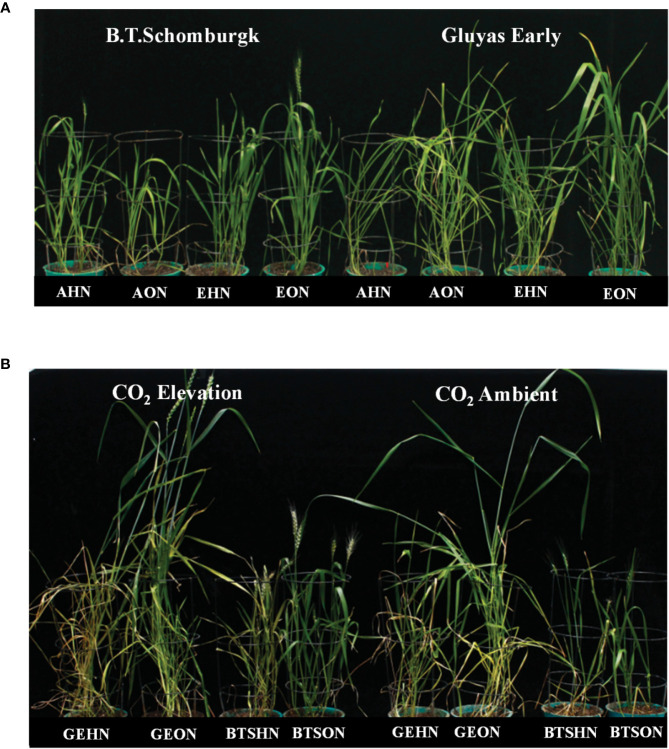
Interactive effect of CO_2_ concentration (CO_2_ EC, 700±10 µl/l, Ambient CO_2_: AC, 400±10 µl/l) and nitrogen availability (High Nitrogen: HN, 20mM nitrate, Optimum Nitrogen: ON, 5mM nitrate) on growth and phenology of wheat seedlings under variable nitrogen supply. **(A)** before flowering **(B)** after flowering in pot grown plants of wheat genotypes Gluyas Early (V1) and BT-Schomburgk (V2).

Plants grown under CE had increased plant heights, among all the treatment combinations, V1 plants receiving HN nutrition were tallest **(****Figure 3D****)**. However, the maximum biomass was observed in V1 plants receiving ON under CE environment **(****Figure 3E****)**. CE decreased the shoot N content of V1 irrespective of N supply, whereas V2 plants did not show decrease in N content. At every treatment combination, V2 plants maintained higher N content than V1 **(****Figure 3G****)**. High nitrogen availability resulted in higher C%, more so under CA conditions **(****Figure 3F****)**. Plants growth under CE showed increase in C/N ratio in both the genotypes, albeit higher C/N ratio was seen in HN in V1 **(****Figure 3H****)**. Observations on various yield parameters were also recorded, however since majority of the V1 plants did not flower under CA conditions, the data were not analyzed for statistical significance. In nutshell, V2 plants performed better in terms of response to CE and N availability. **Figures 4A, B** depict the impact of CE and N levels on plant growth at before flowering and after flowering. Plants grown under CE and receiving HN performed better than their CA counterparts. It can also be noted that CE accelerated phenological processes and resulted in early flowering (as seen in ON treatment of V1 in CE and HN and ON treatments of V2 in CE; **Figure 4B**). High nitrogen supply was toxic to the plants and decreased the various growth parameters, most prominently under CA. CE could partially alleviate the high N induced damages.

### CE Increases the Content of Photosynthetic Pigments

**Figure 5:**
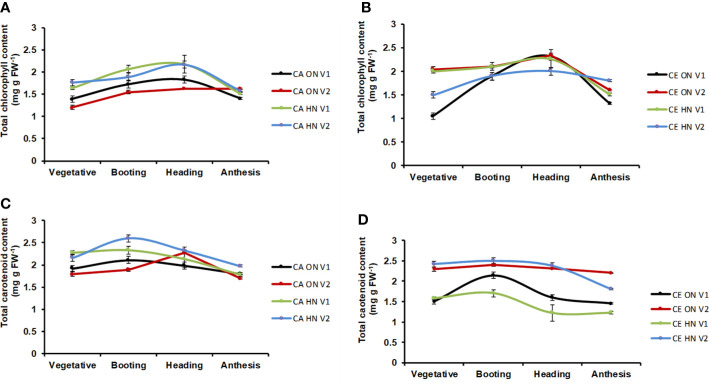
Interactive effect of CO_2_ elevation (CE, 700±10 µl/l, CO_2_ ambient CA 400±10 µl/l) and Optimum Nitrogen (ON: 5mM nitrate) and High Nitrogen (HN: 20mM nitrate) availability on accumulation of photosynthesis pigments **(A, B**: Total chlorophyll content; **C, D**: Total carotenoid content) at different growth stages in pot grown plants of wheat genotypes Gluyas Early (V1) and BT-Schomburgk (V2). Values are mean (±SE) of 3 biological replicates.

Differences in total chlorophyll and carotenoid content (mg g^−1^ DW) were significant at different CO_2_ levels and genotypes. At all stages, total chlorophyll content was higher under CE than CA (9% increase over CA). Chlorophyll content showed a progressive increase from vegetative to anthesis stages and showed a slight decline during anthesis (**Figures 5A, B****)**. Total carotenoid content was always higher under CA than CE (7% increase over CE). In all the treatment combinations carotenoid content showed a progressive increase from vegetative to booting stage and showed a slight decline afterwards **(****Figures 5C, D****)**. Plants receiving ON maintained higher chlorophyll and carotenoid level under CE.

### CE Enhances Accumulation of Soluble Sugar and Starch

**Figure 6:**
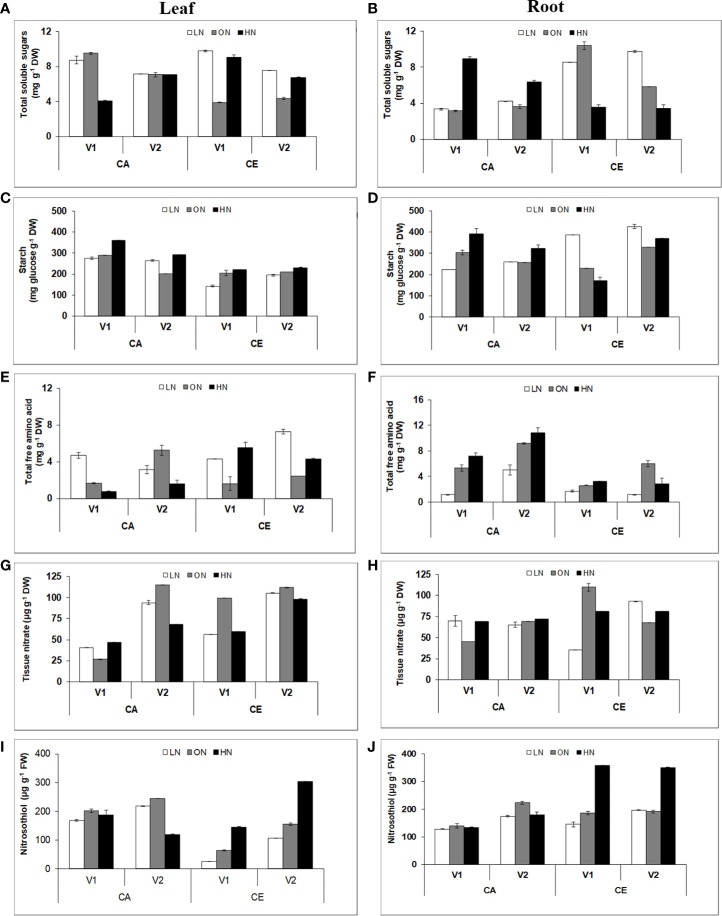
Interactive effect of CO_2_ elevation (CE, 700±10 µl/l, CO_2_ ambient :CA, 400±10µl/l) and nitrogen availability (Low Nitrogen: LN, 0.05mM nitrate, Optimum Nitrogen: ON, 5mM nitrate, High Nitrogen: HN, 20mM nitrate) on content of soluble sugar **(A, B)**, starch **(C, D)**, total free amino acids **(E, F)**, tissue nitrate **(G, H)** and S-nitrosothiols **(I, J)** in leaf and root tissues of 40 days old seedlings of wheat genotypes Gluyas Early (V1) and BT-Schomburgk (V2) raised in hydroponic culture. Values are mean (±SE) of 3 biological replicates.

Plants grown under CE showed increased soluble sugar contents in both leaves and roots **(****Figures 6A, B****)**. However, the changes in root sugar content were manifold and highly significant. There was a significant difference in root sugar content among the genotypes. V1 showed 45% and V2 showed 34% increase in root soluble sugar content under CE than CA. Plants grown under CE showed an increase in starch content of both leaves and roots **(****Figures 6C, D****)**. There were significant differences in shoot and root starch content among the genotypes, with V1 and V2 showing 39 and 20% increase in shoot starch content, and 26 and 34% root starch content, respectively, under CE than CA. Variation in free amino acid content of shoot and root tissues followed a contrasting pattern in both the genotypes **(****Figures 6E, F****)**. There was an overall increase in shoot free amino acid content in the CE treatment w.r.to CA (38–39% increase). In roots there was a consistent decrease in free amino acid content in the CE treatment w.r.to CA (60–80% decrease).

### CE Increases Tissue Nitrate Content

Plants grown under CE showed an increase in tissue nitrate content of both leaves and roots **(****Figures 6G, H****)**. Compared to CA, CE grown plants of both genotypes had significant increase in shoot nitrate content (V1:47%, V2:14%) as well as root nitrate content (V1:17%, V2:18%).

### CE Promotes Accumulation Nitrosothiol and Reactive Oxygen Species

Variations in nitrosothiol content of shoot and root tissues also followed contrasting pattern in both the genotypes. There was an overall decrease in shoot nitrosothiol content in CE treatment w.r.t CA (3–13%) **(****Figures 6I, J****)**. In roots there was consistent increase in nitrosothiol content in CE treatment w.r.t CA.

**Figure 7:**
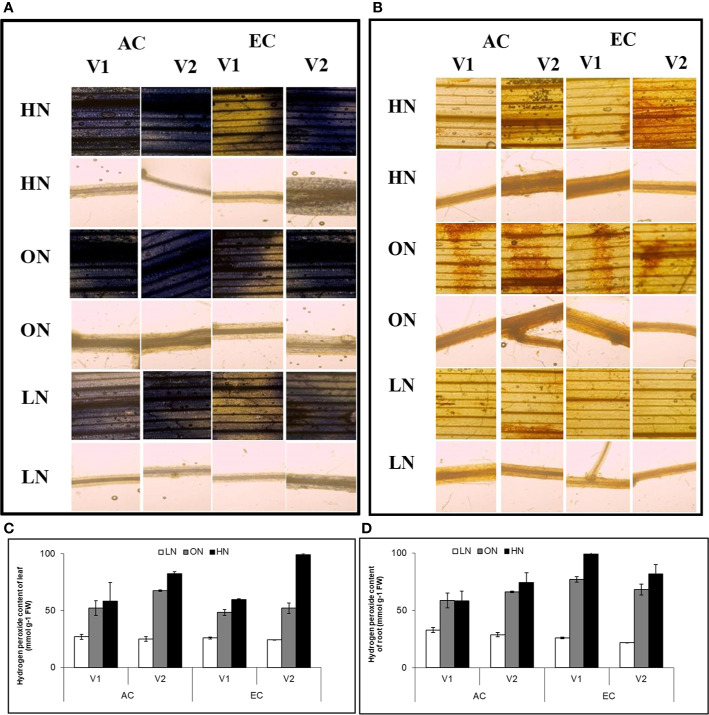
Interactive effect of CO_2_ concentration (CO_2_ Elevation: CE, 700±10 µl/l, CO_2_ Ambient :CA, 400±10µl/l) and nitrogen availability (High Nitrogen: HN, 20mM nitrate, Optimum Nitrogen: ON, 5mM nitrate, Low Nitrogen: LN, 0.05mM nitrate) on **(A)** accumulation of superoxide radicals and **(B)** hydrogen peroxide **(C, D)** content of hydrogen peroxide in leaf and root samples were collected from genotypes after 30 days exposure to treatments. Images are representative from three biological replicates. Valuea are mean (±SE) of 3 biological replicates.

Accumulation of both ROS and hydrogen peroxide were highest in HN, followed by ON treatments in both leaf and root tissues. CE reduced the accumulation of superoxide radicals in genotype V1 and LN treatment of V2, whereas in ON and HN treatments, EC increased superoxide radical levels in V2 roots. The accumulation of hydrogen peroxide was reduced by CE in LN treatment in both the genotypes. Whereas in ON and HN treatments EC increased superoxide radical levels in V2 leaves. In short, the impact of CE on ROS generation and abundance is variable depending on the nitrate supply and genotype (**Figures 7A–D**).

### CE Affects the Activity of N Metabolism Enzymes

**Figure 8:**
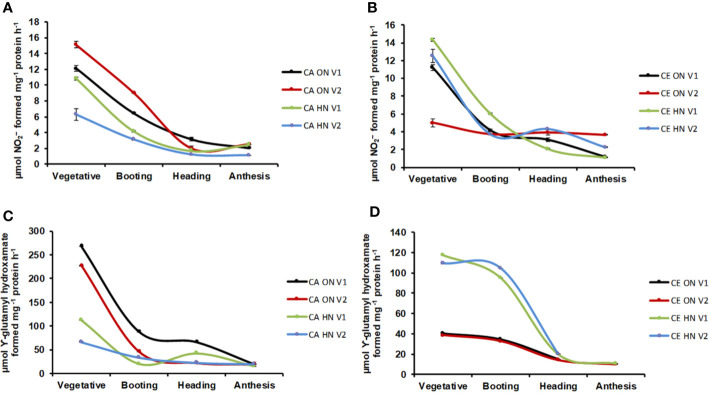
Interavtive effect of CO_2_ elevation (CE, 700±10 µl/l, CO_2_ ambient: CA, 400±10 µl/l) and Optimum Nitrogen (ON: 5mM nitrate) and High Nitrogen (HN: 20mM nitrate) availability on activity of enzymes nitrates reductase (NR, **A, B** and glutamine synthase (GS, **C, D)** at different growth stages in pot grown plants of wheat genotypes Gluyas Early (V1) and BT-Schomburgk (V2). Values are mean (±SE) of 3 biological replicates.

**Figure 9:**
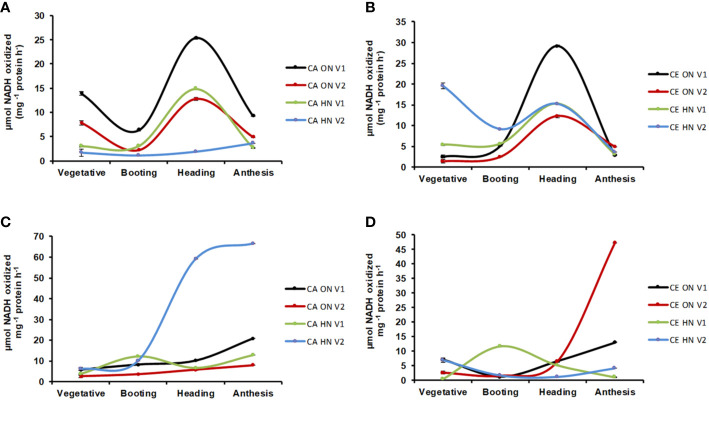
Interactive effect of CO_2_ elevation (CE, 700±10 µl/l, CO_2_ ambient: CA, 400±10 µl/l) and Optimum Nitrogen (ON: 5mM nitrate) and High Nitrogen (HN: 20mM Nitrate) availability on activity of enzymes glutamate synthases (GOGAT, **A, B)** and glutamate dehydrogenase (GDH, **C, D)** at different growth stages in pot grown plants of wheat genotypes Gluyas Early (V1) and BT-Schomburgk (V2). Values are mean (±SE) of 3 biological replicates.

Significant differences in NR activity were observed with various levels of N and CO_2_ at different stages of growth. Across developmental stages, activity of NR was lower under CE than CA (24% decrease over CA). Activity of NR showed a progressive decline from the vegetative to the anthesis stage **(****Figures 8A, B****)**. CE down-regulated shoot NR activity, however, V1 plants receiving HN maintained higher enzyme activity. Glutamine synthetase (GS) activity was significantly different w.r.t N and CO_2_ levels in leaves and roots. GS activity showed an overall decline under CE (by 51%). In all treatment combinations, activity of GS also showed a progressive decline from vegetative to anthesis stages **(****Figures 8C, D****)**. CE down regulated shoot GS activity, however, plants receiving HN maintained higher enzyme activities under CE. Glutamate synthase (GOGAT) activity showed significant differences under various levels of N and CO_2_. GOGAT activity was increased by 20% under CE than CA. Under all the treatments, maximum GOGAT activity was recorded during heading stage **(****Figures 9A, B****)**. Significant difference was observed in NADH-GDH activity with various levels of N and CO_2_. In leaves, CE reduced the NADH-GDH activity by 50%. The highest value of GDH activity was recorded at anthesis stage in all the treatment combinations **(****Figures 9C, D****)**.

### Expression of Genes Involved in Nitrogen Metabolism and Antioxidant Defense

**Figure 10:**
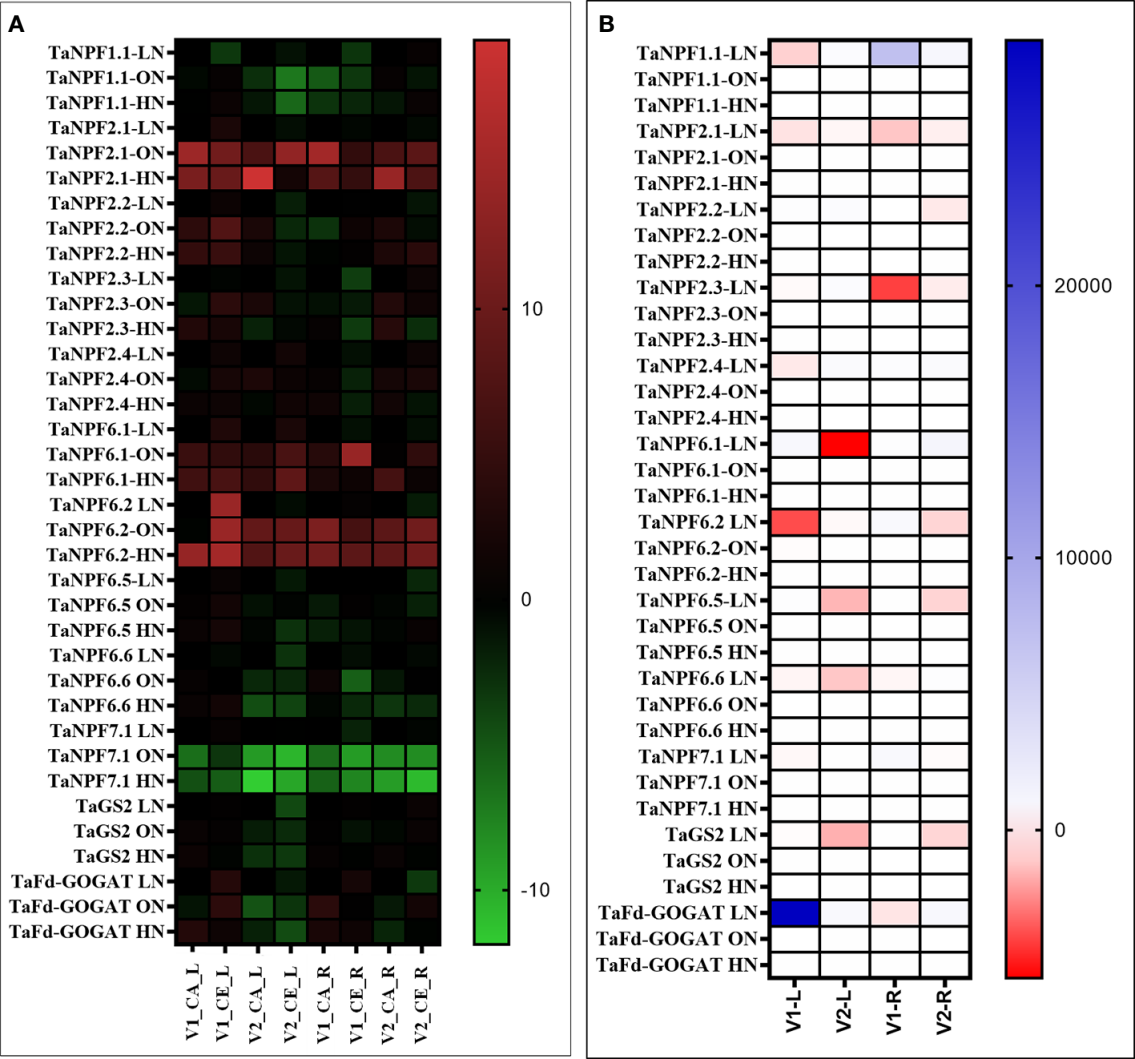
Interactive effect of CO_2_ concentration (CO_2_ Elevation: CE, 700±10 µl/l, CO_2_ Ambient :CA, 400±10 µl/l) and nitrogen availability (Low Nitrogen: LN, 0.05mM nitrate, Optimum Nitrogen: ON, 5mM nitrate, High Nitrogen: HN, 20mM nitrate) on the expression of nitrate uptake and assimilatory genes in leaf and root tissues of wheat genotypes Gluyas Early (V1) and BT-Schomburgk (V2) raised in hydroponic culture. Heat map depicting **(A)** Relative fold (Log2) expression values (w.r.to LN) and **(B)** relative expression (CE/CA). Color-codes are given in respective color scale bars. Leaf and root samples were collected from genotypes after 30 days exposure to treatments.

Plants grown under CE showed reduced the expression of *TaNPF6.1*, *TaNPF6.2*, *TaGS2* and *TaFd-GOGAT* in leaves. Similarly, expression levels of *TaNPF1.1*, *TaNPF2.2*, *TaNPF7.1*, *TaNPF6.1* and *TaNPF6.2* were reduced by CE in leaves (**Figures 10A, B****)**. In roots TaNPF1.1, *TaNPF2.1*, *TaNPF2.3*, *TaNPF6.5*, *TaNPF6.6* were induced by CE. Whereas *TaNPF7.1* was highly expressed in both shoots and roots, albeit the expression was higher in roots. The expression of *TaNPF2.1* was reduced by CE in V2, and up-regulated by CE in V1. In contrast, expression of *TaNPF1.1* was reduced by CE in V1, and up-regulated by CE in V2. The expression of *TaNPF2.2* was reduced by CE in V1, and up-regulated by CE in V2. Expression of *TaNPF2.3* was up regulated by CE in both the genotypes. Expression of *TaNPF6.5* and *TaNPF6.6* were also differentially affected by CE. In case of *TaNPF6.5*, expression was specifically reduced by CE in V2. Photorespiratory ammonia assimilatory genes were down-regulated by CE. Expression of *NPF* genes were differentially regulated by CE, and N availability in different genotypes.

**Figure 11:**
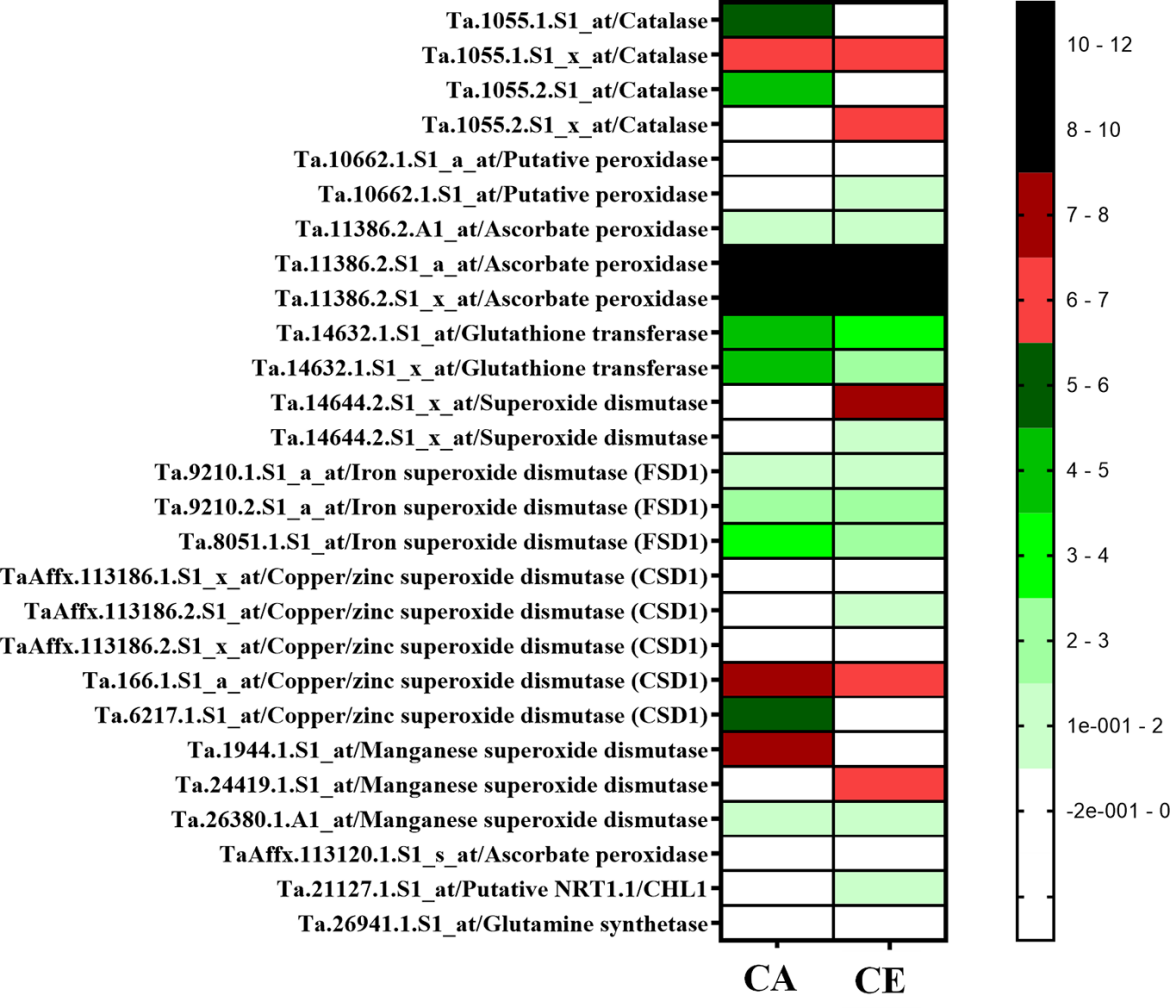
Expression heat map of antioxidant enzyme encoding genes from experiment GSE48620 (study comparing the global gene expression of *Triticum aestivum* cv Nortstar grown either CO_2_ Ambient (CA) or CO_2_ Elevation (CE). Retrieved from publically open database (https://www.ncbi.nlm.nim.gov/gds).

Expression data of antioxidant enzyme encoding genes from experiment GSE48620 (study comparing the global gene expression of *T. aestivum* cv Norstar grown either under CA or CE) was retrieved from publically open database (https://www.ncbi.nlm.nih.gov/gds). Expression response of genes encoding, Catalase, Putative peroxidases, Ascorbate peroxidase, Glutathione transferase, Superoxide dismutases: Iron superoxide dismutase (FSD1), Copper/zinc superoxide dismutase (CSD1), Manganese superoxide dismutase, and wheat homologue of *AtNRT1.1/CHL1*and Glutamine synthetase were analyzed. Expression of Glutathione transferase, Superoxide dismutases: Iron superoxide dismutase (FSD1), Copper/zinc superoxide dismutase (CSD1), Manganese superoxide dismutase was down-regulated by CE, whereas expression of Catalase, Putative peroxidases, Ascorbate peroxidase, *AtNRT1.1/CHL1* and Glutamine synthetase were upregulated by CE (**Figure 11****)**.

## Discussion

Mineral nutrient availability, and nutrient uptake and utilization efficiency of plants determine the long-term responses of the plants to CO_2_ elevation (CE). Higher level of atmospheric CO_2_ enhances photosynthesis as well as growth of plant, and results in acclimation responses leading to higher demand for mineral nutrients. Effective utilization of nutrients or higher nutrient uptake and assimilation may counter the higher nutrient demand in CE condition. With optimum nitrogen (ON) supply, shoot biomass of CE grown plants were highest that that of CA grown plants which may be due to higher leaf area and net photosynthetic rate per unit leaf area of plants grown under CE. Similarly, Bunce (2002) also observed the increase in leaf area of CE grown plants. Exposure to CE improved root growth (Hachiya et al., 2014; Lekshmy et al., 2016), fine-root production (Iversen, 2010; Beidler et al., 2015) but suppressed root length increase (Vicente et al., 2016) irrespective of N availability. CE promoted an increase in total chlorophyll content irrespective of N treatment in hydroponic experiment. In contrast CE reduced carotenoid contents under high N supply in both genotypes. A previous study also reported a reduction in carotenoid content up to 25% in CE grown plants as compared to control plants (Aguera et al., 2006). Interestingly, chlorophyll content remained same for both CA and CE till heading, and started declining fast in CE probably due to remobilization of N to the larger sink.

The oxidative status of leaves was found to be altered in CE grown plants which was also observed in earlier studies performed with different plant species (Cheeseman, 2006; Qiu et al., 2008). CE elevation led to increased production of ROS (Erice et al., 2007) and reduced level of antioxidants and antioxidant enzyme activity (Wustman et al., 2001). In agreement with the previous results, CE could alleviate ROS abundance (Pritchard et al., 2000) in LN and ON conditions, whereas under HN CE enhanced the accumulation of ROS. The response of ROS generation and abundance is variable depending on the nitrate supply and CO_2_ concentration. There were differences in response of the two genotypes w.r.t. abundance of ROS, possibly due to the difference in nitrate uptake and assimilation as shown in **Supplementary Files 1A, B**. Variety V1 has a low nitrate uptake rate, and higher root nitrate assimilation, whereas V2 has high a nitrate uptake capacity and high leaf nitrate assimilation. This implies V2 takes up large amounts of nitrate under HN supply and nitrate ions get assimilated in leaves. As reported earlier, CE enhances nitrate uptake assimilation from LN conditions (Lekshmy et al., 2009; Lekshmy et al., 2013) and inhibits uptake and shoot nitrate assimilation in HN conditions (Lekshmy et al., 2013; Adavi and Sathee, 2019). This could be the reason behind higher ROS abundance in CE at HN supply and which was more pronounced in high NUE genotype (V2). Qui et al. (2008) also reported higher protein carbonization and attributed this to increased H_2_O_2_ which might have transcriptionally upregulated cytosolic *APX1*. The *in silico* gene expression presented in this study, and reported previously (Miyazaki et al., 2004; Li et al., 2006) also indicate that CE up-regulates transcripts associated with redox control, indicative of altered ROS balance.

It is important to take into account the nutrient acquisition and its utilization mechanisms for understanding the response of plant growth and metabolism in CE condition. Plant growth under CE increased the C/N ratio of wheat genotypes. Downstream to nitrate assimilation the ensuing ammonium assimilation for synthesis of amino acid requires carbon skeleton derived from photosynthesis. Enhanced carbon availability due to higher CO_2_ fixation under CE vitalizes the NO_3_^-^ utilization by augmenting the activity and expression of NR, NiR as well as chloroplastic GS (Larios et al., 2004). Aguera et al. (2006) recorded that with increased CO_2_ levels GS activity in plants was up-regulated. But, it was also found that GS activity was decreased in CE grown plants under HN levels, and this could be due to inhibition of nitrate photoassimilation and hence non availability of ammonium (Bloom et al., 2002). Apart from a potential dilution effect, this strongly supports, the hypothesis that the pathway of nitrate assimilation is being inhibited leading to reduction in N content under CE. So, crop species which uptake N in the form of NO_3_^-^ ions found to be competitively disadvantageous in NH_4_^+^ dependent species under CE (Smart et al., 1998).

Reduction in tissue N contents are due to high NUE, hence plant growth is not limited under CE (Zerihun et al., 2000). Coleman et al. (1993) found that in response to CO_2_ elevation, plant N contents were reduced as compared to that of CA plants. Despite the decreases in N on a weight basis in CE, organic N content per plant often increases (Hocking and Meyer, 1991). A part of the percent N reduction under CE may account for the reported reduction in RUBISCO and *vice versa*. Changes in the photorespiratory metabolism also contribute to reduction in N contents as photorespiration is repressed by CE (Conroy and Hocking, 1993). Carbohydrate accumulation was observed in flag leaves and roots under CE (Stitt and Krapp, 1999; Long et al., 2004; Perez et al., 2005) and this accumulation was found to be more pronounced under low N than high N supply in tobacco (Geiger et al., 1999) and durum wheat (Vicente et al., 2015). Exposure of low N grown plants to CE resulted in downregulation of photosynthetic rate, closure of stomata, decrease in transpiration rate, increase in sugar content and decrease in organic N content specifically in photosynthetic tissues, increase in intermediates of Calvin–Benson cycle. LN grown plants also displayed dilution of Rubisco enzyme, decrease in NR activity and transcripts for N metabolism and non-significant change in nitrate uptake (Stitt and Krapp, 1999; Perez et al., 2005; Taub and Wang, 2008; Gutierrez et al., 2013; Vicente et al., 2016).

Du et al. (2016) showed that CE induced production of nitric oxide inhibits NR activity in *Arabidopsis*. Under CE, the level of S-nitrosoglutathione (GSNO), a transient store of NO also increases probably due to the inhibition of S-nitrosoglutathione reductase (GSNOR) by NO. Accumulation of GSNO further suppresses the nitrate uptake by HATS by inhibiting the transcription of HATS (Frungillo et al., 2014). We observed an overall decrease in shoot nitrosothiol content and an increase in root nitrosothiol content in CE treatment. Redistribution of nitrate to developing leaves is a critical step mediated by *NPF1.1* and *NPF1.2* which are potentially involved in xylem-to-phloem transfer (Buchner et al., 2015). Genotype V2 displayed higher expression of *TaNPF1.1* in leaves of CE grown plants, suggesting higher leaf translocation and storage of nitrate, which would sustain higher root nitrate uptake and thus higher NUE in comparison to V1. Transcript abundance of *TaNPF6.1* and *TaNPF6.2* was higher in roots than that in shoots. *TaNPF6.3* showed same levels of transcripts in both shoot and root while *TaNPF6.4* transcript abundance was higher in roots than in shoots. Expression levels of *TaNPF4.1*, *TaNPF6.3* and *TaNPF6.4* was much lower than that of *TaNPF6.1* and *TaNPF6.2* and has similar level of expression in both shoots and roots. In *Arabidopsis*, *NPF6.3* mediated auxin transport determines auxin abundance and lateral root initiation and elongation (Krouk et al., 2010). Gene expression of *TaNPF6.2* and *TaNPF6.3* were responsive to N supply (Buchner and Hawkesford, 2014). It is quite possible that, these two putative auxin transporting *NPFs* are candidates offering wheat a sensitive mode of N responsive of root system architecture. Out of the various *NPF* genes analyzed *TaNPF7.1* showed highest transcript abundance in roots. Expression analysis of Wheat, *NPF* genes were previously described (Buchner and Hawkesford, 2014; Bajgain et al., 2018). *TaNPF6.6*, *TaNPF6.5*, *TaNPF2.1* and *TaNPF2.2* were highly expressed in the shoot (Buchner and Hawkesford, 2014). Expression data of antioxidant enzyme encoding genes from experiment GSE48620 (https://www.ncbi.nlm.nih.gov/gds) (**Figure 11**) indicates a differential regulation of antioxidant defense transcripts by CE. Down-regulation in expression of superoxide scavenging SOD (super oxide dismutase) isoforms, reinstates the enhanced superoxide radical accumulation of CE grown plants.

**Figure 12:**
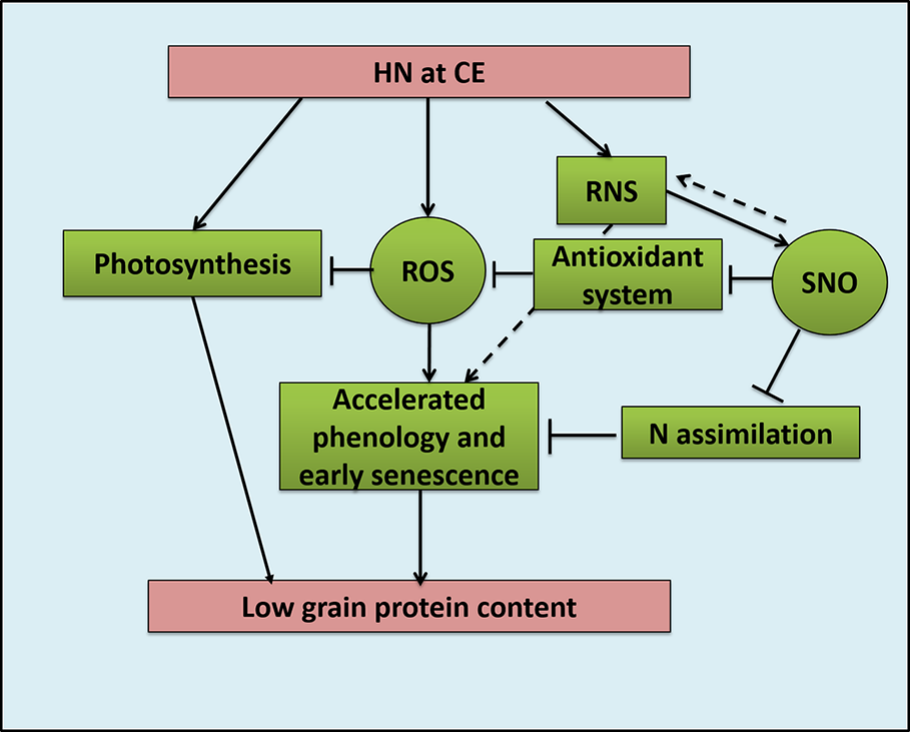
Schematic model representing effects oh High Nitrogen (20mM nitrate) supply under CO_2_ Elevation (700±10 µl/l) condition on Reactive Oxygen Species and Reactive Nitrogen Species balance and crop growth and quality in bread wheat; HN: High Nitrogen, CE: CO_2_ Elavation, ROS: Reactive Oxygen Species, RNS: Reactive Nitrogen Species, SNO: S-nitrosothiol.

Plant growth under CE showed accelerated phenological processes and accelerated senescence mechanisms (**Figure 12**). High nitrogen supply was toxic to the plants and decreased various growth parameters, most prominently under CA. CE could partially alleviate the high N induced damages. Genotype with higher N use efficiency performed better under CE conditions. Previous reports also suggest the acceleration of growth, phenology and senescence by CE in wheat and sunflower (de la Mata et al., 2012: Buchner et al., 2015). As CO_2_ elevation promoted the leaf senescence and thus demonstrates the complex interaction between C/N ratio, soluble sugar level and oxidative status during the leaf ontogeny. The variation in contents of N, C and the resultant alteration in C/N ratio evokes a signaling in response to C/N balance (Zheng, 2009). The alteration in the C/N balance affects senescence progression. Report from Aoyama et al. (2014) showed the role of ubiquitin ligase *ATL31* in leaf senescence to the balance between availability of N and atmospheric CO_2_ in *Arabidopsis*. In *Arabidopsis*, *ATL31* acts in co-ordination with *WRKY53* the key regulator of senescence. The possibility of such a mechanism operating in wheat and other cereal crops can be speculated and should be evaluated in future.

## Conclusion

Our results showed that response of plants to N availability under CE is genotype dependent. Hence N fertilizer regimes need to be revised based on the NUE and responsiveness of the cultivars. Attention also needs to be given to the altered crop phenology under CE, also responsible for the discrepancy in N fertilization requirements. Hence, it is essential to develop cultivars resilient to changing environmental condition, while maintaining yield and food quality food.

## Data Availability Statement

Data is available in the article and **Supplementary Files**. Publicly available datasets were analyzed in this study. This data can be found here: GSE48620.

## Author Contributions

BP and LS conducted the experiments and prepared the first draft. LS designed primers, did the expression profiling, statistical analysis and finalized the figures. HM assisted in growing of plants and taking observations. SA contributed in selection of genotypes and manuscript drafting. LS, SJ, and VC finalized experiments, contributed resources and revised the manuscript.

## Conflict of Interest

The authors declare that the research was conducted in the absence of any commercial or financial relationships that could be construed as a potential conflict of interest.

## References

[B1] Adavi S. B. Sathee L. (2018). Elevated CO_2_ induced production of nitric oxide differentially modulates nitrate assimilation and root growth of wheat seedlings in a nitrate dose-dependent manner. Protoplasma 256 (1), 147–159. 10.1007/s00709-018-1285-2 30032354

[B2] Aguera E. Ruano D. Cabello P. de la Haba P. (2006). Impact of atmospheric CO_2_ on growth, photosynthesis and nitrogen metabolism in cucumber (*Cucumis sativus* L.) plants. J. Plant Physiol. 163, 809–817. 10.1016/j.jplph.2005.08.010 16777528

[B3] Ainsworth E. A. Long S. P. (2005). What have we learned from 15 years of free air CO_2_ enrichment (FACE)? A meta analytic review of the responses of photosynthesis, canopy properties and plant production to rising CO_2_. New Phytol. 165 (2), 351–372. 10.1111/j.1469-8137.2004.01224.x 15720649

[B4] Ainsworth E. A. Rogers A. Leakey A. D. (2008). Targets for crop biotechnology in a future high CO_2_ and high O_3_ world. Plant Physiol. 147 (1), 13–19. 10.1104/pp.108.117101 18443102 PMC2330306

[B5] Aoyama S. Huarancca Reyes T. Guglielminetti L. Lu Y. Morita Y. Sato T. (2014). Ubiquitin ligase ATL31 functions in leaf senescence in response to the balance between atmospheric CO_2_ and nitrogen availability in *Arabidopsis*. Plant Cell Physiol. 55 (2), 293–305. 10.1093/pcp/pcu002 24399238 PMC3913444

[B6] Arai H. Berlett B. S. Chock P. B. Stadtman E. R. (2005). Effect of bicarbonate on iron-mediated oxidation of low-density lipoprotein. Proc. Natl. Acad. Sci. U.S.A. 102:, 10472–10477. 10.1073/pnas.0504685102 16027354 PMC1176232

[B7] Arc E. Galland M. Godin B. Cueff G. Rajjou L. (2013). Nitric oxide implication in the control of seed dormancy and germination. Front. Plant Sci. 4, 346. 10.3389/fpls.2013.00346 24065970 PMC3777103

[B8] Arnon D. II (1949). Copper enzymes in isolated chloroplasts. Polyphenol oxidase in *Beta vulgaris*. Plant Physiol. 24 , 1. 10.1104/pp.24.1.1 16654194 PMC437905

[B9] Asensio J. S. R. Rachmilevitch S. Bloom A. J. (2015). Responses of *Arabidopsis* and wheat to rising CO_2_ depend on nitrogen source and night-time CO_2_ levels. Plant Physiol. 168 (1), 156–163. 10.1104/pp.15.00110 25755253 PMC4424024

[B10] Bajgain P. Russell B. Mohammadi M. (2018). Phylogenetic analyses and in-seedling expression of ammonium and nitrate transporters in wheat. Sci. Rep. 8, 7082. 10.1038/s41598-018-25430-8 29728590 PMC5935732

[B11] Beidler K. V. Taylor B. N. Strand A. E. Cooper E. R. Schonholz M. Pritchard S. G. (2015). Changes in root architecture under elevated concentrations of CO_2_ and nitrogen reflect alternate soil exploration strategies. New Phytol. 205 (3), 1153–1163. 10.1111/nph.13123 25348775

[B12] Bloom A. J. Meyerhoff P. A. Taylor A. R. Rost T. L. (2002a). Root development and absorption of ammonium and nitrate from the rhizosphere. J. Plant Growth Regul. 21 (4), 416–431. 10.1007/s00344-003-0009-8

[B13] Bloom A. J. Smart D. R. Nguyen D. T. Searles P. S. (2002b). Nitrogen assimilation and growth of wheat under elevated carbon dioxide. Proc. Natl. Acad. Sci. 99 (3), 1730–1735. 10.1073/pnas.022627299 11818528 PMC122259

[B14] Bloom A. J. Burger M. Asensio J. S. R. Cousins A. B. (2010). Carbon dioxide enrichment inhibits nitrate assimilation in wheat and Arabidopsis. Science 328 (5980), 899–903. 10.1126/science.1186440 20466933

[B15] Buchner P. Hawkesford M. J. (2014). Complex phylogeny and gene expression patterns of members of the NITRATE TRANSPORTER 1/PEPTIDE TRANSPORTER family (NPF) in wheat. J. Exp. Bot. 65 (19), 5697–5710. 10.1093/jxb/eru231 24913625 PMC4176842

[B16] Buchner P. Tausz M. Ford R. Leo A. Fitzgerald G. J. Hawkesford M. J. (2015). Expression patterns of C and N metabolism related genes in wheat are changed during senescence under elevated CO_2_ in dry land agriculture. Plant Sci. 236, 239–249. 10.1016/j.plantsci.2015.04.006 26025537

[B17] Bunce J. A. (2002). Carbon dioxide concentration at night affects translocation from soybean leaves. Ann. Bot. 90, 399–403. 10.1093/aob/mcf203 12234152 PMC4240403

[B18] Chaitanya K. S. K. Naithani S. C. (1994). Role of superoxide, lipid peroxidation and superoxide dismutase in membrane perturbation during loss of viability in seeds of *Shorea robusta* Gaernt.f. New Phytol. 126, 623–627. 10.1111/j.1469-8137.1994.tb02957.x

[B19] Cheeseman J. M. (2006). Hydrogen peroxide concentrations in leaves under natural conditions. J. Exp. Bot. 57 (10), 2435–2444. 10.1093/jxb/erl004 16766599

[B20] Coleman J. S. McConnaughay K. D. M. Bazzaz F. A. (1993). Elevated CO_2_ and plant nitrogen-use: is reduced tissue nitrogen concentration size-dependent? Oecologia 93 (2), 195–200. 10.1007/BF00317671 28313607

[B21] Conroy J. Hocking P. (1993). Nitrogen nutrition of C_3_ plants at elevated atmospheric CO_2_ concentrations. Physiol. Plant 89 (3), 570–576. 10.1111/j.1399-3054.1993.tb05215.x

[B22] Correia C. M. Pereira J. M. M. Coutinho J. F. Bjorn L. O. Torres-Pereira J. M. (2005). Ultraviolet-B radiation and nitrogen affect the photosynthesis of maize: a Mediterranean field study. Eur. J. Agron. 22 (3), 337–347. 10.1016/j.eja.2004.05.002

[B23] Cotrufo M. F. Ineson P. Scott A. (1998). Elevated CO_2_ reduces the nitrogen concentration of plant tissues. Glob. Change Biol. 4 (1), 43–54. 10.1046/j.1365-2486.1998.00101.x

[B24] de la Mata L. Cabello P. de la Haba P. Aguera E. (2012). Growth under elevated atmospheric CO_2_ concentration accelerates leaf senescence in sunflower (*Helianthus annuus* L.) plants. J. Plant Physiol. 169 (14), 1392–1400. 10.1016/j.jplph.2012.05.024 22818664

[B25] Dhami N. Tissue D. T. Cazzonelli C. II (2018). Leaf age dependent response of carotenoid accumulation to elevated CO_2_ in *Arabidopsis*. Arch. Biochem. Biophys. 647, 67–75. 10.1016/j.abb.2018.03.034 29604257

[B26] Downes M. T. (1978). An improved hydrazine reduction method for the automated determination of low nitrate levels in freshwater. Water Res. 12, 673–675. 10.1016/0043-1354(78)90177-X

[B27] Drake B. G. Gonzalez Meler M. A. Long S. P. (1997). More efficient plants: a consequence of rising atmospheric CO_2_? Annu. Rev. Plant Biol. 48 (1), 609–639. 10.1146/annurev.arplant.48.1.609 15012276

[B28] Du S. T. Zhang Y. S. Lin X. Y. Wang Y. Tang C. X. (2008). Regulation of nitrate reductase by nitric oxide in Chinese cabbage pakchoi (*Brassica chinensis* L.). Plant Cell Environ. 31, 195–204. 10.1111/j.1365-3040.2007.01750.x 18028279

[B29] Du S. Zhang R. Zhang P. Liu H. Yan M. Chen N. (2016). Elevated CO_2_ induced production of nitric oxide (NO) by NO synthase differentially affects nitrate reductase activity in *Arabidopsis* plants under different nitrate supplies. J. Exp. Bot. 67 (3), 893–904. 10.1093/jxb/erv506 26608644

[B30] Erice G. Aranjuelo I. Irigoyen J. J. Sanchez-Diaz M. (2007). Effect of elevated CO_2_, temperature and limited water supply on antioxidant status during regrowth of nodulated alfalfa. Physiol. Plant 130 (1), 33–45. 10.1111/j.1399-3054.2007.00889.x

[B31] Foyer C. H. Noctor G. (2020). Redox Homeostasis and Signaling in a Higher-CO_2_ World. Annu. Rev. Plant Biol. 71, 157–182. 10.1146/annurev-arplant-050718-095955 32442392

[B32] Frungillo L. Skelly M. J. Loake G. J. Spoel S. H. Salgado I. (2014). S-nitrosothiols regulate nitric oxide production and storage in plants through the nitrogen assimilation pathway. Nat. Commun. 5, 5401. 10.1038/ncomms6401 25384398 PMC4229994

[B33] Geiger M. Haake V. Ludewig F. Sonnewald U. Stitt M. (1999). The nitrate and ammonium nitrate supply have a major influence on the response of photosynthesis, carbon metabolism, nitrogen metabolism and growth to elevated carbon dioxide in tobacco. Plant Cell Environ. 22 (10), 1177–1199. 10.1046/j.1365-3040.1999.00466.x

[B34] Gruda N. Bisbis M. Tanny J. (2019). Influence of climate change on protected cultivation: impacts and sustainable adaptation strategies - a review. J. Clean Prod. 225, 481–495. 10.1016/j.jclepro.2019.03.210

[B35] Gutierrez D. Morcuende R. Del Pozo A. Martinez-Carrasco R. Perez P. (2013). Involvement of nitrogen and cytokinins in photosynthetic acclimation to elevated CO_2_ of spring wheat. J. Plant Physiol. 170 (15), 1337–1343. 10.1016/j.jplph.2013.05.006 23747059

[B36] Hachiya T. Sugiura D. Kojima M. Sato S. Yanagisawa S. Sakakibara H. (2014). High CO_2_ triggers preferential root growth of *Arabidopsis thaliana* via two distinct systems under low pH and low N stresses. Plant Cell Physiol. 55 (2), 269–280. 10.1093/pcp/pcu001 24401956 PMC3913443

[B37] Hawkesford M. J. (2012). The diversity of nitrogen use efficiency for wheat varieties and the potential for crop improvement. Better Crops 96 (3), 10–12.

[B38] Hirel B. Le Gouis J. Ney B. Gallais A. (2007). The challenge of improving nitrogen use efficiency in crop plants: towards a more central role for genetic variability and quantitative genetics within integrated approaches. J. Exp. Bot. 58 (9), 2369–2387. 10.1093/jxb/erm097 17556767

[B39] Hiscox J. T. Israelstam G. F. (1979). A method for the extraction of chlorophyll from leaf tissue without maceration. Can. J. Bot. 57 (12), 1332–1334. 10.1139/b79-163

[B40] Hoagland D. R. Arnon D. II (1950). The water-culture method for growing plants without soil. Calif. AES 347, 23–32.

[B41] Hocking P. J. Meyer C. P. (1991). Effects of CO_2_ enrichment and nitrogen stress on growth, and partitioning of dry matter and nitrogen in wheat and maize. Funct. Plant Biol. 18 (4), 339–356. 10.1071/PP9910339

[B42] IPCC (2014). “Summary for Policy makers,” in Climate change 2014: impacts, adaptation, and vulnerability. Part a: global and sectoral aspects. Contribution of working group II to the fifth assessment report of the intergovernmental panel on climate change. Eds. Field C. B. Barros V. R. Dokken D. J. Mach K. J. Mastrandrea M. D. Bilir T. E. Chatterjee M. Ebi K. L. Estrada Y. O. Genova R. C. Girma B. Kissel E. S. Levy A. N. MacCracken S. Mastrandrea P. R. White L. L. (Cambridge: Cambridge University Press), 1–32.

[B43] Iversen C. M. (2010). Digging deeper: fine root responses to rising atmospheric CO_2_ concentration in forested ecosystems. New Phytol. 186 (2), 346–357. 10.1111/j.1469-8137.2009.03122.x 20015070

[B44] Jauregui I. Aparicio Tejo P. M. Avila C. Canas R. Sakalauskiene S. Aranjuelo I. (2016). Root-shoot interactions explain the reduction of leaf mineral content in Arabidopsis plants grown under elevated [CO_2_] conditions. Physiol. Plant 158 (1), 65–79. 10.1111/ppl.12417 26801348

[B45] Kellner J. Houska T. Manderscheid R. Weigel H. J. Breuer L. Kraft P. (2019). Response of maize biomass and soil water fluxes on elevated CO_2_ and drought–from field experiments to process-based simulations. Glob. Change Biol. 25, 2947–2957. 10.1111/gcb.14723 31166058

[B46] Kjeldahl C. (1883). A new method for the determination of nitrogen in organic matter. Anal. Chem. 22, 366. 10.1007/BF01338151

[B47] Klepper L. Flesher D. Hageman R. H. (1971). Generation of reduced nicotinamide adenine dinucleotide for nitrate reduction in green leaves. Plant Physiol. 48 (5), 580–590. 10.1104/pp.48.5.580 16657841 PMC396909

[B48] Krouk G. Lacombe B. Bielach A. Perrine-Walker F. Malinska K. Mounier E. (2010). Nitrate-regulated auxin transport by *NRT1.1* defines a mechanism for nutrient sensing in plants. Dev. Cell. 18 (6), 927–937. 10.1016/j.devcel.2010.05.008 20627075

[B49] Kumar D. Yusuf M. A. Singh P. Sardar M. Sarin N. B. (2014). Histochemical Detection of Superoxide and H_2_O_2_ Accumulation in *Brassica juncea* Seedlings. Bio-protocol 4 (8), e1108. 10.21769/BioProtoc.1108

[B50] Kumari M. Verma S. C. Bhardwaj S. K. (2019). Effect of elevated CO_2_ and temperature on crop growth and yield attributes of bell pepper (*Capsicum annuum* L.). J. Agrometeorol. 21 (1), 1–6.

[B51] Lam S. K. Chen D. Norton R. Armstrong R. Mosier A. R. (2012). Nitrogen dynamics in grain crop and legume pasture systems under elevated atmospheric carbon dioxide concentration: a meta-analysis. Glob. Change Biol. 18, 2853–2859. 10.1111/j.1365-2486.2012.02758.x 24501062

[B52] Large E. B. (1954). Growth stages in cereals. Illustrations of the FEEKES SCALE. Plant Pathol. 3, 128–129.

[B53] Larios B. Aguera E. Cabello P. Maldonado J. M. De La Haba P. (2004). The rate of CO_2_ assimilation controls the expression and activity of glutamine synthetase through sugar formation in sunflower (*Helianthus annuus* L.) leaves. J. Exp. Bot. 55 (394), 69–75. 10.1093/jxb/erh017 14645390

[B54] Leakey A. D. (2009). Rising atmospheric carbon dioxide concentration and the future of C_4_ crops for food and fuel. P. R. Soc Lond. B Biol. 276 (1666), 2333–2343. 10.1098/rspb.2008.1517 PMC2690454 19324804

[B55] Lekshmy S. Jain V. Khetarpal S. Pandey R. Singh R. (2009). Effect of elevated carbon dioxide on kinetics of nitrate uptake in wheat roots. Indian J. Plant Physiol. 14 (1), 16–22.

[B56] Lekshmy S. Jain V. Khetarpal S. Pandey R. (2013). Inhibition of nitrate uptake and assimilation in wheat seedlings grown under elevated CO2. Ind. J. Plant Physiol. 18, 23–29. 10.1007/s40502-013-0010-6

[B57] Lekshmy S. Jain V. Khetarpal S. Verma R. Sailo N. Pandey R. (2016). Influence of elevated carbon dioxide and ammonium nutrition on growth and nitrogen metabolism in wheat. Indian J. Agr. Sci. 86 (1), 25–30.

[B58] Lekshmy S. Jha S. K. (2017). Selection of reference genes suitable for qRT-PCR expression profiling of biotic stress, nutrient deficiency and plant hormone responsive genes in bread wheat. Indian J. Plant Physiol. 22 (1), 101–106.

[B59] Li P. Sioson A. Mane S. P. Ulanov A. Grothaus G. Heath L. S. (2006). Response diversity of *Arabidopsis thaliana* ecotypes in elevated [CO_2_] in the field. Plant Mol. Biol. 62, 593–609. 10.1007/s11103-006-9041-y 16941220

[B60] Li D. Zhang X. Li L. Aghdam M. S. Wei X. Liu J. (2019). Elevated CO_2_ delayed the chlorophyll degradation and anthocyanin accumulation in postharvest strawberry fruit. Food Chem. 285, 163–170. 10.1016/j.foodchem.2019.01.150 30797331

[B61] Loladze I. Nolan J. M. Ziska L. H. Knobbe A. R. (2019). Rising Atmospheric CO_2_ Lowers Concentrations of Plant Carotenoids Essential to Human Health: A Meta Analysis. Mol. Nutr. Food Res. 63 (15), 1801047. 10.1002/mnfr.201801047 31250968

[B62] Long S. P. Ainsworth E. A. Rogers A. Ort D. R. (2004). Rising atmospheric carbon dioxide: plants FACE the future. Annu. Rev. Plant Biol. 55, 591–628. 10.1146/annurev.arplant.55.031903.141610 15377233

[B63] Long S. P. Ainsworth E. A. Leakey A. D. Nosberger J. Ort D. R. (2006). Food for thought: lower than expected crop yield stimulation with rising CO_2_ concentrations. Science 31, 1928–1921. 10.1126/science.1114722 16809532

[B64] Luo Y. Su B. Currie W. S. (2004). Progressive nitrogen limitation of ecosystem responses to rising atmospheric carbon dioxide. Bioscience 54, 731–739. 10.1641/0006-3568(2004)054[0731:PNLOER]2.0.CO;2

[B65] McCready R. M. Guggolz J. Silviera V. Owens H. S. (1950). Determination of starch and amylose in vegetables. Anal. Chem. 22 (9), 1156–1158. 10.1021/ac60045a016

[B66] Mhamdi A. Noctor G. (2016). High CO_2_ primes plant biotic stress defenses through redox-linked pathways. Plant Physiol. 172 (2), 929–942. 10.1104/pp.16.01129 27578552 PMC5047113

[B67] Miyazaki S. Fredricksen M. Hollis K. C. Poroyko V. Shepley D. Galbraith D. W. (2004). Transcript expression profiles of *Arabidopsis thaliana* grown under controlled conditions and open-air elevated concentrations of CO_2_ and O_3_. Field Crops Res. 90, 47–59. 10.1016/j.fcr.2004.07.010

[B68] Mohanty B. Fletcher J. S. (1980). Ammonium influence on nitrogen assimilating enzymes and protein accumulation in suspension cultures of Paul’s Scarlet rose. Physiol. Plant. 48 (3), 453–459. 10.1111/j.1399-3054.1980.tb03285.x

[B69] Myers S. S. Zanobetti A. Kloog I. Huybers P. Leakey A. D. B. Bloom A. J. (2014). Increasing CO_2_ threatens human nutrition. Nature 510, 139–142. 10.1038/nature13179 24805231 PMC4810679

[B70] Pan T. Ding J. Qin G. Wang Y. Xi L. Yang J. (2019). Interaction of Supplementary Light and CO_2_ Enrichment Improves Growth, Photosynthesis, Yield, and Quality of Tomato in Autumn through Spring Greenhouse Production. Horticult. Sci. 54 (2), 246–252. 10.21273/HORTSCI13709-18

[B71] Pastore M. A. Lee T. D. Hobbie S. E. Reich P. B. (2019). Strong photosynthetic acclimation and enhanced water use efficiency in grassland functional groups persist over 21 years of CO_2_ enrichment, independent of nitrogen supply. Glob. Change Biol. 25, 3031–3044. 10.1111/gcb.14714 31148322

[B72] Perez P. Morcuende R. del Molino I. M. Martıinez-Carrasco R. (2005). Diurnal changes of Rubisco in response to elevated CO_2_, temperature and nitrogen in wheat grown under temperature gradient tunnels. Environ. Exp. Bot. 53 (1), 13–27. 10.1016/j.envexpbot.2004.02.008

[B73] Pritchard S. G. Ju Z. van Santen E. Qiu J. Weaver D. B. Prior S. A. (2000). The influence of elevated CO_2_ on the activities of antioxidant enzymes in two soybean genotypes. Aust. J. Plant Physiol. 27, 1061–10685. 10.1071/PP99206

[B74] Pucher G. W. Leavenworth C. S. Vickery H. B. (1948). Determination of starch in plant tissues. Anal. Chem. 20 (9), 850–853. 10.1021/ac50100a004

[B75] Qiu Q. S. Huber J. L. Booker F. L. Jain V. Leakey A. D. Fiscus E. L. (2008). Increased protein carbonylation in leaves of *Arabidopsis* and soybean in response to elevated [CO_2_]. Photosynth. Res. 97 (2), 155. 10.1007/s11120-008-9310-5 18506594

[B76] Rachmilevitch S. Cousins A. B. Bloom A. J. (2004). Nitrate assimilation in plant shoots depends on photorespiration. Proc. Natl. Acad. Sci. U.S.A. 101 (31), 11506–11510. 10.1073/pnas.0404388101 15272076 PMC509230

[B77] Rogers A. Fischer B. U. Bryant J. Frehner M. Blum H. Raines C. A. (1998). Acclimation of photosynthesis to elevated CO_2_ under low nitrogen nutrition is affected by the capacity for assimilate utilization. Perennial ryegrass under free air CO_2_ enrichment. Plant Physiol. 118, 683–689. 10.1104/pp.118.2.683 9765554 PMC34844

[B78] Sadasivam S. Manikam A. (1996). Biochemical methods for Agricultural Sciences. New Delhi: New age international (P) Limited, Publishers, 193–194.

[B79] Sircelj H. Tausz M. Grill D. Batic F. (2005). Biochemical responses in leaves of two apple tree cultivars subjected to progressing drought. J. Plant Physiol. 162 (12), 1308–1318. 10.1016/j.jplph.2005.01.018 16425449

[B80] Smart D. R. Ritchie K. Bloom A. J. Bugbee B. B. (1998). Nitrogen balance for wheat canopies (*Triticum aestivum*) grown under elevated and ambient CO_2_ concentrations. Plant Cell Environ. 21 (8), 753–763. 10.1046/j.1365-3040.1998.00315.x 11543217

[B81] Stitt M. Krapp A. (1999). The molecular physiological basis for the interaction between elevated carbon dioxide and nutrients. Plant Cell Environ. 22, 583–622. 10.1046/j.1365-3040.1999.00386.x

[B82] Taub D. R. Wang X. (2008). Why are nitrogen concentrations in plant tissues lower under elevated CO_2_? A critical examination of the hypotheses. J. Integr. Plant Biol. 50 (11), 1365–1374. 10.1111/j.1744-7909.2008.00754.x 19017124

[B83] Tausz-Posch S. Borowiak K. Dempsey R. W. Norton R. M. Seneweera S. Fitzgerald G. J. (2013). The effect of elevated CO_2_ on photochemistry and antioxidative defence capacity in wheat depends on environmental growing conditions – A FACE study. Environ. Exp. Bot. 88, 81–92. 10.1016/j.envexpbot.2011.12.002

[B84] Terrer C. Vicca S. Stocker B. D. Hungate B. A. Phillips R. P. Reich P. B. (2018). Ecosystem responses to elevated CO_2_ governed by plant soil interactions and the cost of nitrogen acquisition. New Phytol. 217, 507–522. 10.1111/nph.14872 29105765

[B85] Vicente R. Perez P. Martinez-Carrasco R. Usadel B. Kostadinova S. Morcuende R. (2015). Quantitative RT-PCR platform to measure transcript levels of C and N metabolism-related genes in durum wheat: transcript profiles in elevated (CO_2_) and high temperature at different levels of N supply. Plant Cell Physiol. 56 (8), 1556–1573. 10.1093/pcp/pcv079 26063390

[B86] Vicente R. Perez P. Martinez Carrasco R. Feil R. Lunn J. E. Watanabe M. (2016). Metabolic and transcriptional analysis of durum wheat responses to elevated CO_2_ at low and high nitrate supply. Plant Cell Physiol. 57 (10), 2133–2146. 10.1093/pcp/pcw131 27440546

[B87] Wustman B. A. Oksanen E. Karnosky D. F. Noormets A. Isebrands J. G. Pregitzer K. S. (2001). Effects of elevated CO_2_ and O_3_ on aspen clones varying in O_3_ sensitivity: can CO_2_ ameliorate the harmful effects of O_3_? Environ. Pollut. 115 (3), 473–481. 10.1016/S0269-7491(01)00236-6 11789927

[B88] Xu G. Fan X. Miller A. J. (2012). Plant nitrogen assimilation and use efficiency. Annu. Rev. Plant Biol. 63, 153–182. 10.1146/annurev-arplant-042811-105532 22224450

[B89] Zerihun A. Gutschick V. P. Bassirirad H. (2000). Compensatory roles of nitrogen uptake and photosynthetic N use efficiency in determining plant growth response to elevated CO_2_: evaluation using a functional balance model. Ann. Bot. 86 (4), 723–730. 10.1006/anbo.2000.1234

[B90] Zhang Y. Drigo B. Bai S. H. Menke C. Zhang M. Xu Z. (2017). Biochar addition induced the same plant responses as elevated CO_2_ in mine spoil. Environ. Sci. Pollut. R. 25 (2), 1460–1469. 10.1007/s11356-017-0574-1 29090446

[B91] Zheng Z. L. (2009). Carbon and nitrogen nutrient balance signaling in plants. Plant Signal. Behav. 4 (7), 584–591. 10.4161/psb.4.7.8540 19820356 PMC2710548

[B92] Zhu C. Ziska L. Zhu J. Zeng Q. Xie Z. Tang H. (2012). The temporal and species dynamics of photosynthetic acclimation in flag leaves of rice (*Oryza sativa*) and wheat (*Triticum aestivum*) under elevated carbon dioxide. Physiol. Plant 145, 395–405. 10.1111/j.1399-3054.2012.01581.x 22268610

[B93] Ziska L. (2008). Three year field evaluation of early and late 20^th^ century spring wheat cultivars to projected increases in atmospheric carbon dioxide. Field Crop Res. 108 (1), 54–59. 10.1016/j.fcr.2008.03.006

