# Supplementary material for: CO2 Elevation Accelerates Phenology and Alters Carbon/Nitrogen Metabolism vis-à-vis ROS Abundance in Bread Wheat
Source: Front Plant Sci. 2020 Jul 17;11:1061. doi: 10.3389/fpls.2020.01061 (PMC7379427; doi:10.3389/fpls.2020.01061)
Supplement: Supplementary file 1 [file DataSheet_1.docx]

**Supplementary file 1a. Rate of nitrate uptake (µmol NO_3_^-^ g^-1^ FW h^-1^) in Gluyas Early (V1) and B.T.Schomburgk (V2) seedlings after incubation in nitrate concentrations 0.06mM, 0.6mM and 60mM for 2 hours**

|  | **V1** | **V2** |
| --- | --- | --- |
| **0.06** | 2.2±0.1 | 2.55±0.12 |
| **0.6** | 10.33±0.67 | 14.19±1.21 |
| **60** | 1772.3±22 | 2533.4±14 |

**Supplementary file 1b. Comparison of leaf and root nitrate reductase activity (µmol NO_2_^-^ reduced g^-1^ FW h^-1^) of twenty days old seedlings of Gluyas Early (V1) and B.T.Schomburgk (V2) seedlings grown with 5mM nitrate supply for 15 days under hydroponic culture.**

|  | **V1** | **V2** |
| --- | --- | --- |
| **Leaf** | 0.07±0.01 | 0.14±0.01 |
| **Root** | 0.1±0.007 | 0.05±0.001 |

**Supplementary file 2. Representative images showing set up used for experiment I (trays) (a) and experiment II (pots) (b, c). Wheat genotypes were grown inside growth chambers maintained at CO_2_ Ambient (CA_,_ 400±10 µl/l) or CO_2_ Elevation (CE_,_ 700±10 µl/l) conditions and different nitrate levels. V1 –Gluyas early, V2-B.T.Schomburgk**

**
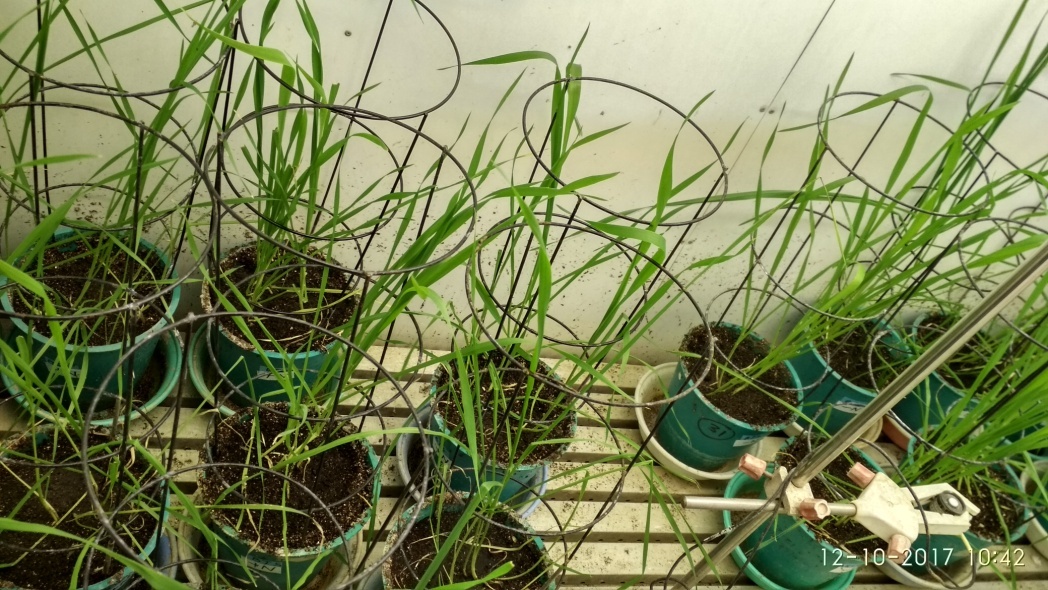

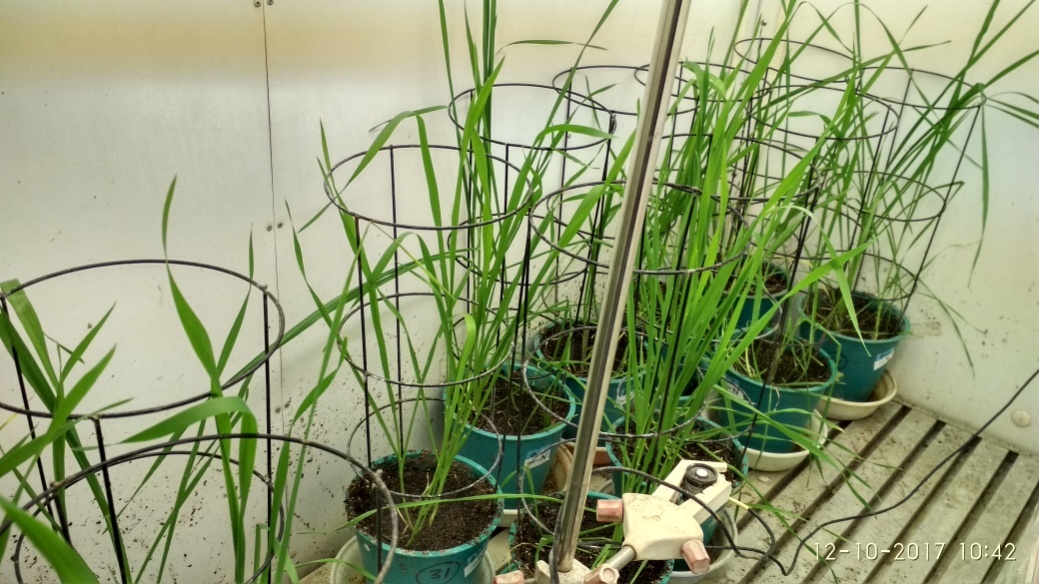

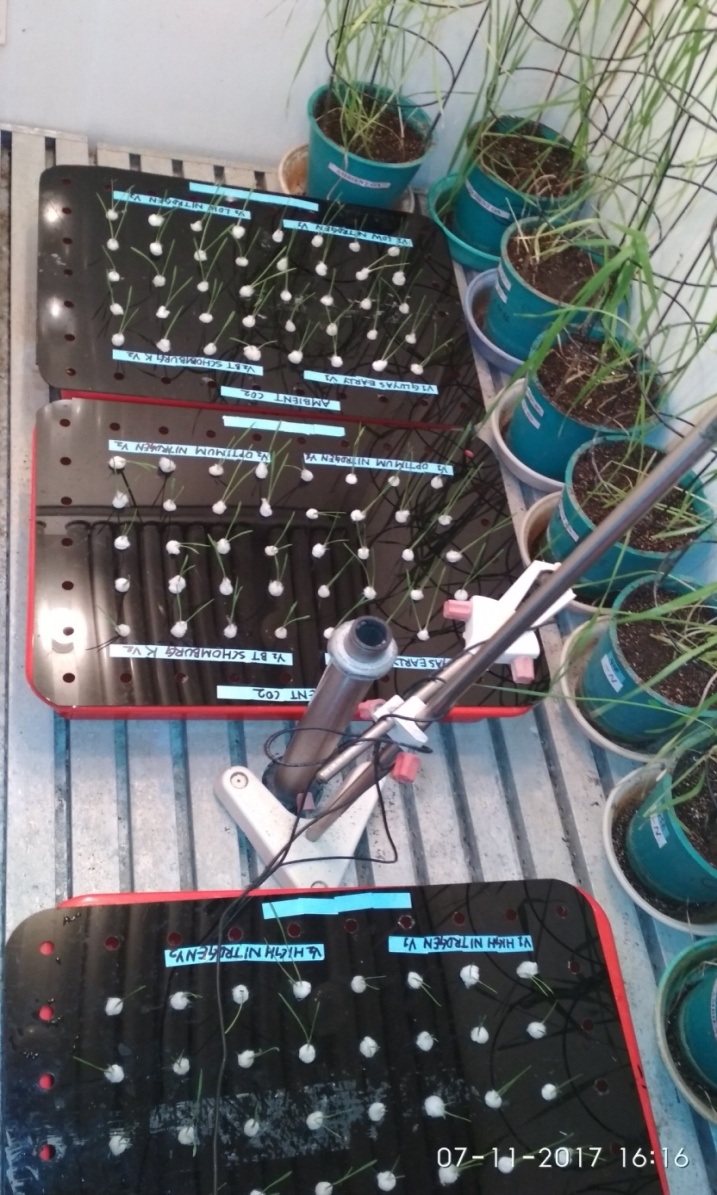
**

**c**

**b**

**a**

**Supplementary file 3. List of primes used to study qRT-PCR expression of nitrate transporters gene**

| **Sl. No.** | **Gene Id** | **Primer Name** | **Sequence** |
| --- | --- | --- | --- |
| **1** | TraesCS3A02G304400 | *TaNPF1.1 -F* | TCCTGTTCACGTCGTTCCT |
| **2** |  | *TaNPF1.1 -R* | CCAGCACGGTGTAGTACCA |
| **3** | TraesCS5A02G004400 | *Ta TaNPF2.1 -F* | CAGGACCTGGACGAGGGT |
| **4** |  | *Ta TaNPF2.1 -R* | CGTCATCGAGCCCAACATC |
| **5** | TraesCS5A02G037900 | *Ta TaNPF2.2 -F* | CTACGCGAGCGGTCTAA |
| **6** |  | *Ta TaNPF2.2 -R* | GACGACGTTCACTGCGG |
| **7** | TraesCS2A02G045500 | *Ta TaNPF2.3 -F* | GAAGGGAGGGTGGACCTATT |
| **8** |  | *Ta TaNPF2.3 -R* | CAGCTTTCTTTGAGCTTTCGTC |
| **9** | TraesCS3A02G418700 | *Ta TaNPF2.4 -F* | CACAAGCTGGTTGACAGATG |
| **10** |  | *Ta TaNPF2.4 -R* | TCTGCTTCCGCCTCTTCC |
| **11** | TraesCS6A02G263500 | *TaNPF7.1-F* | GGAGTGTCGGAAGTGATGA |
| **12** |  | *TaNPF7.1-R* | ATGGACATCATGCACAGCG |
| **13** | TraesCS7A02G301700 | *TaNPF6.1-F* | CGGATTCTTCGTCAGCTCG |
| **14** |  | *TaNPF6.1-R* | GTAGAGCTCGCCCTTGTTG |
| **15** | TraesCS1A02G210900 | *TaNPF6.2 -F* | TCACGGTGTTCCTGCTCAT |
| **16** |  | *TaNPF6.2 -R* | CCTTGTGCACGATGGTTACT |
| **17** | TraesCS1A02G031300 | *TaNPF6.5 -F* | GGCAGCTGGACTTCTTCAT |
| **18** |  | *TaNPF6.5 -R* | CAAGGCGGCTGTGATTGAT |
| **19** | TraesCS5A02G409600 | *TaNPF6.6 -F* | GGACCGTCGCCTCAATG |
| **20** |  | *TaNPF6.6 -R* | AGACAAGGCCCGTTCCTAC |
| **21** | TraesCS2A02G130600 | *Ta Fd-GOGAT-F* | GGTGCCACCCAGCGAAGA |
| **22** |  | *Ta Fd-GOGAT-R* | GCTCGTTTCCAGAAGATGCCTTG |
| **23** | TraesCS2A02G50040 | *TaGS2-F* | ACACTGAGCATGCGCGAAG |
| **24** |  | *TaGS2-R* | AGCTGTCTCGTGTAGCCCT |
